# Cross-cultural adaptation and validation of the Infant Feeding Style Questionnaire in Brazil

**DOI:** 10.1371/journal.pone.0257991

**Published:** 2021-09-30

**Authors:** Jéssica Pedroso, Muriel Bauermann Gubert

**Affiliations:** 1 Postgraduate Program in Human Nutrition, Center for Epidemiological Studies in Health and Nutrition - NESNUT, University of Brasilia, Brasilia, Distrito Federal, Brazil; 2 Department of Nutrition, University of Brasilia, Brasilia, Distrito Federal, Brazil; Iranian Institute for Health Sciences Research, ISLAMIC REPUBLIC OF IRAN

## Abstract

We cross-culturally adapted and validated the Infant Feeding Style Questionnaire (IFSQ) in Brazil. The cross-cultural adaptation and content validity assessment was conducted in five steps: translation, synthesis of translations, back-translation, evaluation by experts, and pre-test. To evaluate reliability, construct validity, and floor and ceiling effects, interviews were conducted with 465 mother-infant pairs at Primary Health Centers in the Federal District, Brazil. The mothers answered the Brazilian Portuguese version of the IFSQ (IFSQ-Br), which evaluated four feeding styles (laissez-faire, pressuring, restrictive, and responsive) from 9 sub-constructs. The indulgent style was not evaluated due to time limitation. We performed reliability analysis using Cronbach’s alpha coefficient and construct validity was evaluated through Confirmatory Factor Analysis. Higher means were found in the sub-constructs of the responsive and restrictive styles. The IFSQ-Br presented adequate reliability (α = 0.73) with values for the Cronbach’s alpha coefficient of the sub-constructs ranging from 0.42 to 0.75. In the Confirmatory Factor Analysis, the final models presented good fit, with the Comparative Fit Indices (CFI) ranging from 0.86 to 1.0 and the Root-Mean Squared Error of Approximation (RMSEA) between 0.0 and 0.09. The IFSQ-Br was shown to be a valid and reliable questionnaire to evaluate maternal feeding beliefs and behaviors in Brazil. Future studies should evaluate the psychometric properties of the indulgent style and include mother-infant pairs from different cultural contexts in Brazil.

## Introduction

Childhood obesity is a worldwide public health problem that increases the risk of chronic diseases and psychological problems in childhood, as well as the chance of children remaining obese in adulthood [1–4]. Among the risk factors for childhood obesity is a diet rich in ultra-processed foods, with a high amount of sugar, fats, and sodium [1,5,6]. The first two years of life are characterized as a window of opportunity to prevent childhood obesity and is a period when preferences and eating habits are formed [6–8].

At this stage of life, children depend on their caregivers to feed them. They are primarily responsible for determining the social context of meals as well as the quantity and the quality of the food offered to the child [7,8]. Feeding styles are behaviors and attitudes that parents use in the context of feeding children [9,10] and can be positively or negatively related to food consumption, weight gain, nutritional status, and eating behavior [1,9,10].

The responsive feeding style is currently recommended and is characterized by a reciprocity between children and their caregivers, who recognize and respond to the child’s hunger and satiety cues, promoting autonomy and development [1,4,7,10,11]. This parenting style has been associated with healthier eating, better regulation of food consumption, and consequently, with the prevention of obesity [1,7,10]. On the other hand, nonresponsive feeding styles lack reciprocity, and there may be a lack of interaction between caregivers and the child during feeding (as in the laissez-faire style), excessive control by the parents (as in restrictive and pressuring styles), or complete control of feeding by the child (as in the indulgent style) [4,7,9]. Nonresponsive styles can have negative consequences, impair the innate regulation of hunger and satiety, favor unhealthy eating habits, and increase the risk of being overweight [7].

To study this important theme, the Infant Feeding Style Questionnaire (IFSQ) was developed in the United States. It is a self-reporting instrument that assesses, based on the beliefs and behaviors of caregivers of infants and young children, five feeding styles: laissez-faire, restrictive, pressuring, responsive, and indulgent [9]. This instrument was initially developed and validated with low-income African-American mothers and its Spanish version was validated with Latino families [9,12]. The IFSQ has been used widely in the United States and in some studies conducted in other countries, such as England [2,13] and the Netherlands [14]. These include studies that evaluate maternal and nonmaternal caregivers feeding styles [15] as well as the association of feeding styles with parental eating behaviors [10], parental acculturation [13,16], duration of breastfeeding [17], and early introduction of solid foods [2,18]. The instrument has also been used to evaluate interventions that seek to prevent childhood obesity and/or encourage an adequate complementary feeding [3,14,19].

Despite the importance of research that evaluates feeding styles in the first years of life, this theme has been studied in Brazil only in children over 2 years of age through the Child Feeding Questionnaire (CFQ), which assesses, among other aspects the use of controlling child feeding strategies, such as restriction and pressuring, among parents of children between 2 and 11 years old [20–22].

Due to the importance of studying infant feeding styles, there is a need for reliable instruments that have been adapted and validated for the Brazilian population to evaluate these aspects in children under two years of age, especially in the phase of introducing complementary feeding, when the infant has the first feeding experiences [6]. Thus, the present study aimed to cross-culturally adapt and validate the IFSQ in Brazil (IFSQ-Br).

## Material and methods

### Infant Feeding Style Questionnaire (IFSQ)

The IFSQ assesses feeding beliefs and behaviors of mothers of infants and young children. It consists of 83 items and evaluates, from 13 sub-constructs, five infant feeding styles: laissez-faire (sub-constructs: attention and diet quality), restrictive (sub-constructs: amount and diet quality), pressuring (sub-constructs: finish, cereal, and soothing), responsive (sub-constructs: satiety and attention) and indulgent (sub-constructs: permissive, coaxing, soothing, and pampering). Each item is assessed using a 5-point Likert scale, with different response options for items that assess beliefs (disagree, slightly disagree, neutral, slightly agree, agree) and behaviors (never, seldom, half of the time, most of the time, always).

The 11 items referring to the laissez-faire style reflect an absence of limits about the quantity and quality of food, as well a lack of interaction with the infant in the context of meals. In the restrictive style (11 items), parents can adopt behaviors such as limiting the amount of food consumed by the infant and their access to unhealthy foods. In the pressuring style (17 items), parents can use food to soothe and are more concerned with increasing the amount of food consumed by their infant. The responsive style (12 items) represents greater attention to the infant’s hunger and satiety cues, as well as monitoring their diet’s quality. In the indulgent style (32 items), there are no established limits for the quantity or quality of the food that the infant consumes [9].

The IFSQ was originally developed by Thompson et al. [9], who verified an internal reliability for the sub-constructs ranging from 0.75 to 0.95, factor loadings from 0.22 to 1.00, Comparative Fit Indices (CFI) from 0.95 to 1.00, and Root Mean Squared Error of Approximation (RMSEA) from 0.00 to 0.13. The Spanish version exhibited reliability coefficients ranging from 0.28 to 0.83, CFI from 0.82 to 1.00, and RMSEA from 0.00 to 0.31 [12].

### Cross-cultural adaptation of IFSQ-Br and content validity

**Fig 1:**
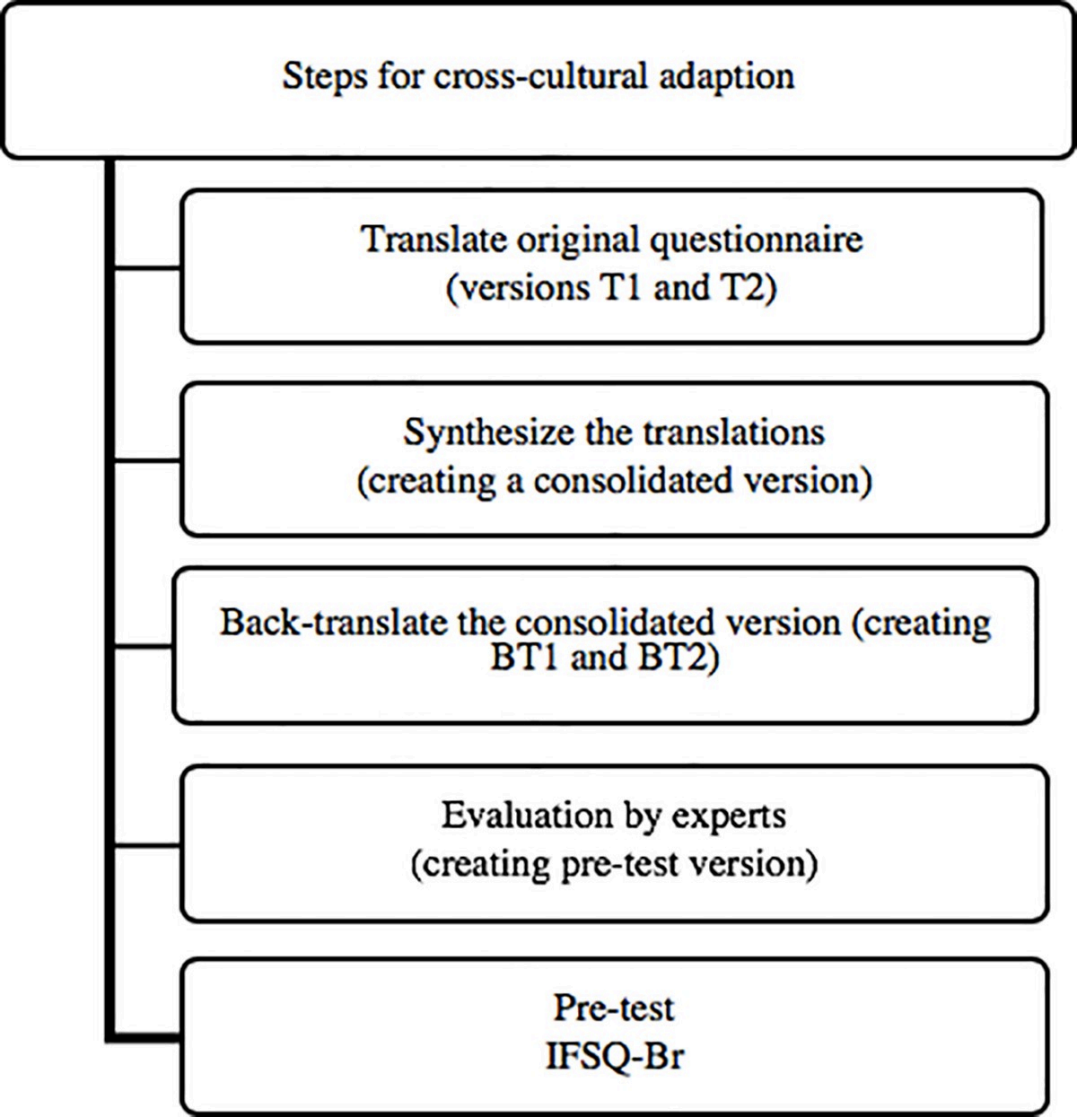
Steps for the cross-cultural adaptation and evaluation of the content validity of the Infant Feeding Style Questionnaire. Federal District, Brazil. 2018.

The process of cross-cultural adaptation of the IFSQ to the Brazilian version (IFSQ-Br) and evaluation of the content validity was conducted in five steps (Fig 1):

- Step 1 (Translation of the original questionnaire): the original questionnaire in English was translated into Brazilian Portuguese by two Brazilian translators, independently, creating versions T1 and T2.
- Step 2 (Synthesis of the translations): the researchers synthesized the two translations, generating a consolidated version.
- Step 3 (Back translation of the consolidated version): back translation into English by two translators, native English speakers, fluent in Brazilian Portuguese, resulting in versions BT1 and BT2. The researchers checked the back translations (BT1 and BT2) looking for inconsistencies in comparison with the original version (IFSQ).
- Step 4 (Evaluation by experts): Twenty experts in nutrition or public health, Brazilian and fluent in English, were invited via email to participate in this step. A link to an online questionnaire available on the Survey Monkey® platform was sent to each of the experts. The questionnaire included the original version (IFSQ items in English) and the consolidated version in Brazilian Portuguese. Below each question, a box was available in which the experts could write any comments or disagreements. In addition, some specific questions were asked. For these questions, previous suggestions were included, and the experts could answer whether or not they agreed with the suggested modification or indicate another option. We suggested: a definition of the age group referred to as infant (under 1 year old) and toddler (1 to 3 years) at the beginning of the questionnaire, since these age categories are not usual in Brazil; the replacement of the word “cereals” with “thickening cereals”, since in Brazil the word “cereals” relate to breakfast cereals; the inclusion of examples of fast food, junk food and thickening cereals; the addition of the option “I don’t know” to items that evaluate beliefs; and the replacement of “finish breast milk” with “empty the entire breast” in item PR3. In addition, we asked about the best translation for “keep track” in items LF6 and LF7. Based on the experts’ opinions, a pre-test version of the questionnaire was created.
- Step 5 (Pre-test): according to the number of participants suggested by Beaton et al. (2000), the questionnaire was applied to 30 mothers in 9 primary health centers (PHC) of the Federal District [23]. The inclusion criteria consisted of adult mothers (≥ 20 years old) whose infants were between 6 and 12 months old and attended immunization or growth and development monitoring sessions in the PHC. We excluded infants with pathologies that could directly or indirectly influence food consumption and infant feeding styles (seizures, cardiopathy, syphilis, mononucleosis, jaundice, glucose 6‐phosphate dehydrogenase—G6PD—deficiency, phenylketonuria, bronchitis, asthma, meningitis, and anemia).

The questionnaire was applied by the first author who verified whether the mother had questions about any item. The IFSQ-Br included the modifications made. All mothers completed the whole questionnaire in the pre-test and no values were missing.

We obtained permission from the corresponding author of the original questionnaire [9] to conduct the cross-cultural adaptation and validation of the IFSQ.

### Assessment of the reliability, construct validity, and floor and ceiling effects of the IFSQ-Br

To assess reliability, construct validity, and floor and ceiling effects, the final version of the IFSQ-Br was applied in 20 PHC of the Federal District to 465 mothers of infants between 6 and 12 months old, which meets the sample size recommended by Terwee et al. of a minimum of 7 individuals per item [24]. The same inclusion and exclusion criteria as the pre-test were used.

The mothers answered a questionnaire that included the IFSQ-Br and sociodemographic data: family per capita monthly income (up to ¼ Brazilian minimum wage/> ¼ and ≤ ½ Brazilian minimum wage/> ½ Brazilian minimum wage); maternal age (20 to 35 years/≥ 35 years), educational level (incomplete or complete elementary school/incomplete or complete high school/incomplete or complete college education and above), race (white/non-white) and marital status (married or living with a partner/single or divorced or separated); infant age (6 to 7 months/8 to 9 months/10 to 12 months), and sex. Items related to the indulgent style were not included due to time limitation, as in the validation among Latino families [12].

Initially, we used SPSS software version 20.0 for descriptive analysis and to assess the reliability of the scale and of each sub-construct using the Cronbach’s alpha coefficient. Cronbach’s alpha values ≥0.6 were considered as indicative of an adequate internal consistency [25].

Confirmatory Factor Analysis (CFA) was conducted using the AMOS software version 22.0 to obtain the factorial loads and the model fit of each sub-construct, as performed by Thompson et al. and Wood et al. [9,12]. The following criteria established a priori were used to assess model fit: Comparative Fit Indices (CFI) ≥ 0.95; Root Mean Squared Error of Approximation (RMSEA) ≤ 0.06 (CI 90% upper limit ≤ 0.08); Chi-Square to Degree of Freedom ratio (CMIN/DF) <3.0; Chi-squared test; and Bayesian Information Criteria (BIC) (for model comparison, with smaller values indicating better fit) [26,27]. The Asymptotic Distribution Free (ADF) method was employed to estimate the parameters. When the initial model had poor fit, modifications were made to improve it. The modifications followed the theoretical model, excluding variables with low factor loads and/or including error covariance between similar items. We did not conduct data imputation, as no variable had more than 10% missing values. No missing values were found for the items of the IFSQ, but we did not consider the analysis items responded to as “I don’t know/does not apply.” For the CFA, we excluded pairs in which mothers responded to at least one item of the sub-construct “I don’t know/does not apply”.

We evaluated floor and ceiling effects by calculating the frequencies of the lowest (disagree or never) or the highest scores (agree or always) on the Likert scale for each item. A frequency lower then 15% on the lowest or highest scores was considered acceptable [24].

### Ethical considerations

This study was approved by the Research Ethics Committee of the School of Health Sciences at University of Brasília (67069417.0.0000.0030) and by Research Ethics Committee of the Foundation of Education and Research in Health Sciences (67069417.0.3001.5553). All mothers agreed to participate in the study and signed the Informed Consent Form.

## Results

### Cross-cultural adaptation and content validity

The step of translation, synthesis of translations, and back-translation occurred satisfactorily as proposed in the methodology (S1 Table).

The questionnaire was evaluated by six experts and modifications were made from the responses. The definition of ages of infants and toddlers at the beginning of the IFSQ-Br was unanimously recommended. All the experts agreed that it was important to add examples of thickened cereals and junk food, thus, corn starch and other flours were given for thickened cereal and processed snacks, candies, and stuffed cookies for junk food. In addition, 83.3% of them indicated that it was important to include examples of fast food, which were selected based on the examples provided in the National School Health Survey Questionnaire (food served in snack bars, hot dog stands, pizzeria…) [28]. The “I don’t know/don’t apply” answer option was also included in the items that referred to beliefs, which presented answer options on a Likert scale, varying between disagree and agree. Item PR3 was modified according to suggestions and in items LF6 and LF7, “keep track” was translated as suggested by most experts (50.0%) (S1 Table). In addition, examples of sugary drinks were also included according to the food consumption markers proposed by the Brazilian Ministry of Health (e.g., boxed juice, powdered juice, boxed coconut water, fruit juice with added sugar, soft drinks) [29].

The pre-test verified good understanding of all items of the questionnaire, with observations by at least one mother on only 5 (PR3, PR5, RP3, RP11, LF1) of the 83 items. To improve understanding, minor modifications were made.

### Assessment of reliability, construct validity, and floor and ceiling effects

We found that 41.6% of the women participating in the study had a family per capita monthly income lower than or equal to half Brazilian minimum wage, 61.9% did not have access to higher education, 72.0% were married or living with a partner, and 24.5% were white. Most of the infants were male (54.0%) and 40.4% were between 6 and 7 months old (Table 1).

**Table 1:** Descriptive analysis of the 465 mother-infant pairs participating in the Brazilian Portuguese version of the Infant Feeding Style Questionnaire (IFSQ-Br) validation. Federal District, Brazil. 2018.

Variables	n	%
**Family per capita monthly income^a^**		
Up to ¼ minimum wage	70	16.0
> ¼ and ≤ ½ minimum wage	112	25.6
> ½ minimum wage	255	58.4
**Maternal age**		
< 35 years	356	76.6
≥ 35 years	109	23.4
**Maternal educational level**		
Incomplete or complete elementary school	64	13.7
Incomplete or complete high school	224	48.2
Incomplete or complete college education and above	177	38.1
**Maternal race**		
Non-white	351	75.5
White	114	24.5
**Maternal marital status**		
Married/living with a partner	335	72.0
Single/divorced/separated	130	28.0
**Infant’s age**		
6 to 7 months	188	40.4
8 to 9 months	160	34.4
10 to 12 months	117	25.2
**Infant’s Sex**		
Female	214	46.0
Male	251	54.0

^a^Minimum monthly wage in the year 2018 = R$ 954.00 equivalent to US$ 238.

For the 9 sub-constructs evaluated in the IFSQ-Br, the highest averages were in the sub-constructs of the responsive style (satiety = 4.51; attention = 4.05) and in the sub-constructs of the restrictive style (amount = 4.19; diet quality = 4.64). The lowest averages were observed in the sub-construct diet quality (1.24) of the laissez-faire style and in the sub-constructs soothing (2.24) and cereal (2.23) of the pressuring style (Table 2).

**Table 2:** Mean, standard deviation, and Cronbach’s alpha coefficient for each sub-construct of Infant Feeding Style Questionnaire. Federal District, Brazil. 2018.

Style (subcontract)	Mean	DP	Cronbach’s alpha coefficient^c^
Laissez-faire (attention)	2.58	0.88	0.42
Laissez-faire (diet quality) ^a^	1.24	0.41	0.46
Pressuring (finish)	3.12	0.82	0.62
Pressuring (cereal)	2.23	1.19	0.69
Pressuring (soothing)	2.24	1.15	0.70
Restrictive (amount)	4.19	1.02	0.67
Restrictive (diet quality) ^b^	4.64	0.61	0.75
Responsive (satiety)	4.51	0.51	0.49
Responsive (attention)	4.05	0.90	0.58

^a^ Items LF6, LF7, LF8, and LF9 were recoded because they are negatively related to the Laissez-faire style.

^b^ Items RS5 and RS6 were recoded because they are negatively related to the Restrictive style.

^c^ Cronbach’s alpha coefficient calculated for the initial model of each sub-construct.

The questionnaire had an overall adequate reliability (α = 0.73) with the values of the Cronbach’s alpha coefficient of the sub-constructs varying from 0.42 to 0.75. Six of the nine sub-constructs exhibited values close to or greater than 0.60, and the sub-constructs of the restrictive and pressuring styles had the highest values (Table 2).

The CFA results found a better fit of the laissez-faire/attention sub-construct model after the removal of item LF4 (I think it is okay to prop an infant’s bottle), which had a low factor load (RMSEA = 0.00; CFI = 1.00). The laissez-faire/diet quality sub-construct did not present a good model fit (RMSEA = 0.05; CFI = 0.66). Items LF10 (A toddler should be able to eat whatever s/he wants for snacks) and LF11 (A toddler should be able to eat whatever s/he wants when eating out at a restaurant) were then excluded, and an error covariance between items LF8-9 (I make sure (child) does not eat sugary food like candy, ice cream, cakes or cookies; I make sure (child) does not eat junk food like potato chips, Doritos and cheese puffs) was included, improving the model (RMSEA = 0.00; CFI = 1.00) (Table 3).

**Table 3:** Model fit indices from the Confirmatory Factor Analysis for each sub-construct of the Brazilian Portuguese version of the Infant Feeding Style Questionnaire (IFSQ-Br). Federal District, Brazil. 2018.

Model	Model detail	Chi-square	CMIN/DF	RMSEA (90% CI)	CFI	BIC
**Laissez-faire (attention)**	Initial model (5 items)	11.78*	2.36	0.06 (0.01–0.10)	0.86	71.79
	4-item model (without item LF4)	0.44	0.22	0.00 (0.00–0.06)	1.00	48.45
**Laissez-faire (diet quality)**	Initial model (6 items)	18.99*	2.11	0.05 (0.02–0.08)	0.66	92.67
	4-item model (without items LF10 and LF11) and with error covariance between items LF8-9	0.83	0.83	0.00 (0.00–0.12)	1.00	56.09
**Pressuring (finish)**	Initial model (8 items)	63.36*	3.17	0.08 (0.06–0.10)	0.71	157.53
	7-item model (without item PR5) and with error covariance between items PR2-4 and PR7-8	28.67*	2.39	0.06 (0.03–0.09)	0.88	122.85
**Pressuring (cereal)**	Initial model (5 items)	52.88*	10.58	0.17 (0.13–0.22)	0.82	110.63
	5-item model with error covariance between items PR12-13 and PR14-15	6.86	2.29	0.06 (0.00–0.13)	0.99	76.15
**Pressuring (soothing)**	Initial model (4 items)	5.82	2.91	0.06 (0.00–0.13)	0.97	54.87
**Restrictive (amount)**	Initial model (4 items)	26.07*	13.03	0.16 (0.11–0.22)	0.76	75.14
	4 -item model with error covariance between items RS1-2	4.54*	4.54	0.09 (0.02–0.18)	0.96	59.74
**Restrictive (diet quality)**	Initial model (7 items)	28.88*	2.06	0.05 (0.02–0.07)	0.72	114.81
	6-item model (without item RS5) and with error covariance between items RS7-8 and RS9-10	5.99	0.86	0.00 (0.00–0.05)	1.00	91.92
**Responsive (satiety)**	Initial model (7 items)	29.16*	2.08	0.05 (0.02–0.07)	0.54	115.03
	5-item model (without RP5 and RP7)	8.33	1.67	0.04 (0.00–0.08)	0.86	69.66
**Responsive (attention)**	Initial model (5 items)	19.02*	3.80	0.08 (0.04–0.12)	0.90	80.42
	4 item model (without item RP12) and with error covariance between items RP8-9	1.93	1.93	0.04 (0.00–0.14)	0.99	57.19

The pressuring/soothing sub-construct model had a good initial fit and no modifications were necessary (RMSEA = 0.06, CFI = 0.97). The pressuring/finish sub-construct model had a better fit after the elimination of item PR5 (Insist re-try new food refused at same meal) and the addition of an error covariance between items PR2-4 (If (child) seems full, encourage to finish anyway; Try to get (child) to eat even if not hungry) and PR7-8 (Important for toddler finish all food on his/her plate; Important for infant finish all milk in his/her bottle) (RMSEA = 0.06, CFI = 0.88). In the pressuring/cereal sub-construct, the inclusion of error covariance between PR12–13 (Cereal in bottle helps infant sleep thru the night; Putting cereal in bottle good b/c helps infant feel full) and PR14–15 (An infant <6 mo needs more than formula or breastmilk to be full; An infant <6 mo needs more than formula or breastmilk to sleep through the night) improved overall model fit (RMSEA = 0.06, CFI = 0.99) (Table 3).

For the restrictive/amount sub-construct model, the inclusion of covariance between the errors of items RS1-2 (I carefully control how much (child) eats; I am very careful not to feed (child) too much) improved the fit of the model (RMSEA = 0.09, CFI = 0.96). The restrictive/diet quality model showed a good model fit with the exclusion of item RS5 (I let (child) eat fast food), which had a low factor load and with the inclusion of an error covariance between items RS7-8 (A toddler should never eat fast food; An infant should never eat fast food) and RS9-10 (A toddler should never eat sugary food like cookies; A toddler should never eat junk food like chips) (RMSEA = 0.00, CFI = 1.00) (Table 3).

The responsive/satiety sub-construct model initially had a poor fit (RMSEA = 0.05, CFI = 0.54), which was improved by dropping items RP5 (I allow (child) to eat when s/he is hungry) and RP7 (Child knows when hungry, needs to eat) that had a low factor load (RMSEA = 0.04, CFI = 0.86). In the model of the responsive/attention sub-construct, the variable RP12 (Important to help or encourage a toddler to eat) was removed due to its low factor load and an error covariance was added between RP8-9 (Talk to (child) to encourage to drink formula/breastmilk; Talk to (child) to encourage him/her to eat), improving model fit (RMSEA = 0.04, CFI = 0.99) (Table 3).

After the modification, all models had lower values of BIC, chi-square, and CMIN/DF (Table 3). Better factor loads are observed in relation to the initial models, varying between 0.22 and 0.89 (Table 4).

**Table 4:** Floor ceiling effect and factor loads of the initial and final models from the Confirmatory Factor Analysis for each sub-construct of the Brazilian Portuguese version of the Infant Feeding Style Questionnaire (IFSQ-Br). Federal District, Brazil. 2018.

Items per style (sub-construct)	n	Lowest score n (%)	Highest score n (%)	Factorial load of original model	Factorial load of final model
**Laissez-faire (attention)**	404				
LF 1		161 (39.9)	155 (38.4)	0.43 ^a^	0.33^a^
LF 2		242 (59.9)	45 (11.1)	0.48*	0.52*
LF 3		197 (48.8)	60 (14.9)	0.45*	0.46*
LF 4		38 (9.4)	319 (79.0)	0.22*	-
LF 5		219 (72.0)	80 (19.8)	0.29*	0.30*
**Laissez-faire (diet quality)**	464				
LF 6		4 (0,9)	420 (90.5)	0.10 ^a^	0.56 ^a^
LF 7		38 (8.2)	363 (78.2)	0.06	0.29
LF 8		11 (2.4)	401 (86.4)	1.77	0.50
LF 9		6 (1,3)	419 (90,3)	0.34	0.22
LF 10		457 (98.5)	6 (1.3)	-0.01	-
LF 11		434 (93.5)	10 (2.2)	0.07	-
**Pressuring (finish)**	360				
PR 1		38 (10.6)	252 (70.0)	0.65 ^a^	0.63 ^a^
PR 2		239 (66.4)	45 (12.5)	0.41*	0.44*
PR 3		139 (38.6)	160 (44.4)	0.58*	0.63*
PR 4		238 (66.1)	37 (10.3)	0.31*	0.29*
PR 5		130 (36.1)	136 (37.8)	0.09	-
PR 6		58 (16.1)	248 (68.9)	0.25*	0.23*
PR 7		44 (12.2)	254 (70.6)	0.46*	0.33*
PR 8		161 (44.7)	129 (35.8)	0.55*	0.45*
**Pressuring (cereal)**	322				
PR 11		171 (53.1)	90 (28.0)	0.48 ^a^	0.59 ^a^
PR 12		150 (46.6)	142 (44.1)	0.69*	0.49*
PR 13		132 (41.0)	147 (45.7)	0.83*	0.57*
PR 14		226 (70.2)	80 (24.8)	0.48*	0.44*
PR 15		251 (78.0)	61 (18.9)	0.42*	0.38*
**Pressuring (soothing)**	460				
PR 16		169 (36.7)	86 (18.7)	0.53^a^	0.53^a^
PR 17		239 (52.0)	117 (25.4)	0.74*	0.74*
PR 18		324 (70.4)	71 (15.4)	0.70*	0.70*
PR 19		265 (57.6)	73 (15.9)	0.48*	0.48*
**Restrictive (amount)**	461				
RS 1		44 (9.5)	334 (72.5)	0.76 ^a^	0.51 ^a^
RS 2		43 (9.3)	358 (77.7)	0.64*	0.39*
RS 3		44 (9.5)	374 (81.1)	0.58*	0.61*
RS 4		128 (27.8)	270 (58.6)	0.54*	0.69*
**Restrictive (diet quality)**	463				
RS 5		424 (91.6)	3 (0.6)	0.04 ^a^	-
RS 6		397 (85.7)	7 (1.5)	0.22	0.31*
RS 7		36 (7.8)	371 (80.1)	0.38	0.44*
RS 8		11 (2.4)	437 (94.4)	0.24	0.37*
RS 9		53 (11.4)	339 (73.2)	0.70	0.74*
RS 10		34 (7.3)	381 (82.3)	0.79	0.89*
RS 11		12 (2.6)	418 (90.3)	0.51	0.52*
**Responsive (satiety)**	461				
RP 1		26 (5.6)	374 (81.1)	0.77 ^a^	0.74 ^a^
RP 2		9 (2.0)	381 (82.6)	0.33*	0.40*
RP 3		151 (32.8)	205 (44.5)	0.22*	0.24*
RP 4		3 (0.7)	426 (92.4)	0.27*	0.29*
RP 5		4 (0.9)	439 (95.2)	0.09	-
RP 6		27 (5.9)	404 (87.6)	0.41*	0.36*
RP 7		16 (3.5)	403 (87.4)	0.07	-
**Responsive (attention)**	464				
RP 8		142 (30.6)	269 (58.0)	0.54^a^	0.39 ^a^
RP 9		69 (14.9)	346 (74.6)	0.87*	0.55*
RP 10		168 (36.2)	217 (46.8)	0.46*	0.62*
RP 11		41 (8.8)	357 (76.9)	0.35*	0.40*
RP 12		3 (0.6)	451 (97.2)	0.17	-

^a^ Parameter set to 1.

* p <0.05.

We observed floor effect in approximately half (52.9%) of the items of the IFSQ, and the frequencies ranged from 0.6% to 98.5%. The ceiling effect was verified on the majority (84.3%) of the items, and the frequencies ranged from 0.6% to 97.2% (Table 4).

## Discussion

The sub-constructs of the responsive and restrictive styles had the highest scores, as observed in previous studies [9,10,12,17]. Responsiveness is very important because it plays a role in preventing childhood obesity, as it stimulates the self-regulation of children’s energy intake based on respecting hunger and satiety cues [1,14]. On the other hand, restriction has been associated in previous studies with an increase in weight gain in the first years of life and a greater risk of overweight [11]. However, it is noteworthy that in relation to the restriction/diet quality subconstruct, the Dietary Guidelines for Brazilian children under 2 years of age does not recommend that ultra-processed foods, sugar, or foods containing sugar be offered to children in this age group [6]. Thus, as these infants were between 6 and 12 months old, a higher score in this sub-construct could reflect greater maternal knowledge about the recommendations. However, this theme was not evaluated in the present study, and we recommend that future studies assess the association between parenting styles and knowledge about infant feeding recommendations.

Previous studies have also observed lower Cronbach’s alpha values in the laissez-faire sub-constructs [12,16]. For the sub-constructs of the responsive style, Wood et al. [12], in a validation study with a Latino families, found higher values than those of the IFSQ-Br (α > 0.60). The values obtained for the sub-constructs of the pressuring and restrictive styles of the IFSQ-Br were similar to those found by Wood et al. [12], with Cronbach’s alpha values close to or above 0.60, showing adequate internal consistency [12]. Thompson et al., assessed the internal consistency of the 13 sub-constructs of the IFSQ in a study with African-American mothers and found a greater range (α = 0.33–0.89) in comparison with the IFSQ-Br (α = 0.42–0.75) [30].

According to the CFA results, the sub-constructs showed good model fit after the modifications, indicating that the IFSQ-Br is valid for assessing infant feeding styles in Brazil. This is the first IFSQ validation outside the United States, where the questionnaire was developed and validated initially in English with low-income African-American mothers by Thompson et al. and later in Spanish with Latino families by Wood et al. [9,12]. Thompson et al. also found adequate model adjustments for all sub-constructs. However, Wood et al. observed low reliability and poor model fit for the laissez-faire style, which was not validated in Spanish. Both studies also required modifications to the questionnaire in the CFA validation process [9,12].

In our study, the subconstruct LF (diet quality) presented non-significant factor loadings. Thompson et al. [9] also found items with non-significant factorial loading in the LF (diet quality) subconstruct for children six months of age or older, and Wood et al. [12] did not validate the LF construct due to its model fit and reliability results. The items with the lowest factor loadings (<0.3) and, therefore, the lowest contribution to the total variance of the subconstruct were found in the subconstructs LF (diet quality), Pressuring (finish), and Responsive (satiety): LF7 (I keep track of how much food (child) eats), LF9 (I make sure (child) does not eat junk food like potato chips, Doritos and cheese puffs), PR4 (Try to get (child) to eat even if not hungry), PR6 (Praise after each bite to encourage finish food), RP3 (I let (child) decide how much to eat), and RP4 (I pay attention when (child) seems to be telling me that s/he is full or hungry). Thompson et al. [9] also found the lowest factor loadings in the LF (diet quality) subconstruct; however, adequate values were found in Pressuring (finish) and Responsive (satiety) subconstructs. Therefore, we suggest that the LF (diet quality) subconstruct, as well as the items with low factor loadings in the Pressuring (finish) (PR4, PR6) and Responsive (satiety) (RP3, RP4) subconstructs should be further explored and refined in qualitative studies based on the experience of the population of interest.

In addition, as conducted in the previous validation studies [9,12], to improve model fit we included error covariance between similar worded items. Although this represents item redundancy, the items evaluate practices or beliefs related to different foods (e.g., RS9 A toddler should never eat sugary food like cookies; RS10 A toddler should never eat junk food like chips), age ranges (e.g., RS7 A toddler should never eat fast food; RS8 An infant should never eat fast food), aspects (e.g., RS1 I carefully control how much (child) eats; RS2 I am very careful not to feed (child) too much), or aims (e.g., PR14 An infant <6 mo needs more than formula or breastmilk to be full; PR15 An infant <6 mo needs more than formula or breastmilk to sleep through the night). Thus, as the exclusion of these items would eliminate a dimension that the other item does not contemplate, the items were not excluded from the questionnaire.

Significant ceiling and floor effects (when the participants tend to choose the extreme options of the Likert scale items) were verified on most IFSQ items. Ceiling and floor effects can limit the distinction between subjects with the lowest or highest scores [24]. In addition, although there is not a consensus regarding the type of changes that a responsive instrument should identify, the floor and ceiling effects can affect the questionnaire responsiveness, making it difficult to identify changes on feeding styles throughout time [24,31–33]. However, to our knowledge, this is the first study that evaluates the IFSQ ceiling and floor effects, as well as the infant feeding styles in Brazil, which does not allow comparisons with previous studies [9,12]. We suggest that the significant ceiling and floor effects can be due to the length of the questionnaire and the type of scale used. Therefore, we recommend that future studies explore further the psychometric properties of the IFSQ and the strategies to reduce ceiling and floor effects, such as changing the Likert scale description or including new items that will have results near to the middle of the Likert scale [32].

Among the limitations of the present study, convenience sample stands out, which limits the generalization of results to populations with different characteristics from the mothers-infant pairs that attend the PHC of the Brazilian Unified Health System in the Federal District. Therefore, we recommend that the questionnaire is applied in a more heterogeneous population, in different Brazilian regions, with different cultures and sociodemographic characteristics in Brazil. In addition, maternal characteristics that can influence feeding styles (e.g., mother’s parity) were not included, and we recommend that future studies consider these aspects.

Despite the limitations, this study is very relevant, supporting the validity and reliability of the IFSQ-Br to measure infant feeding styles. Future studies should evaluate the psychometric properties of the IFSQ-Br, including the indulgent style, since the 32 items related to this construct were not applied with the whole sample due to time limitation. We also recommend that further validation studies investigate the concurrent validity, the common factor bias, and the measurement invariance of the scale.

In conclusion, the results suggest that the IFSQ-Br is a valid and reliable questionnaire to evaluate feeding styles in Brazil and can be used in a range of studies to assess caregivers’ feeding beliefs and behaviors, to evaluate factors associated with feeding styles, as well as the impact on infants’ nutritional status and diet.

## Supporting information

S1 Table Results of the steps for the cross-cultural adaptation of the Infant Feeding Style Questionnaire (IFSQ-Br). Federal District, Brazil. 2018.
(DOCX)

S1 Dataset Validation of the Infant Feeding Style Questionnaire dataset. (XLSX)

S1 Questionnaire Items from the Brazilian Portuguese version of the Infant Feeding Style Questionnaire (IFSQ-Br). (DOCX)

## References

[1] Heller RL, Mobley AR. Instruments assessing parental responsive feeding in children ages birth to 5 years: A systematic review. Appetite. 2019;138: 23–51. doi: 10.1016/j.appet.2019.03.006 30853452

[2] Rogers SL, Blissett J. Infant temperament, maternal feeding behaviours and the timing of solid food introduction. Matern Child Nutr. 2019;15: e12771. doi: 10.1111/mcn.12771 30560584 PMC7198933

[3] Fiks AG, Gruver RS, Bishop-Gilyard CT, Shults J, Virudachalam S, Suh AW, et al. A Social Media Peer Group for Mothers To Prevent Obesity from Infancy: The Grow2Gether Randomized Trial. Child Obes. 2017;13: 356–368. doi: 10.1089/chi.2017.0042 28557558 PMC5647509

[4] Hurley KM, Cross MB, Hughes SO. A Systematic Review of Responsive Feeding and Child Obesity in High-Income Countries. J Nutr. 2011;141: 495–501. doi: 10.3945/jn.110.130047 21270360 PMC3040906

[5] Costa CS, Rauber F, Leffa PS, Sangalli CN, Campagnolo PDB, Vitolo MR. Ultra-processed food consumption and its effects on anthropometric and glucose profile: A longitudinal study during childhood. Nutr Metab Cardiovasc Dis. 2019;29: 177–184. doi: 10.1016/j.numecd.2018.11.003 30660687

[6] Ministério da Saúde. Guia Alimentar para crianças brasileiras menores de 2 anos. Brasília; 2019.

[7] Pérez-Escamilla R, Segura-Pérez S, Lott M. Feeding Guidelines for Infants and Young Toddlers. 2017. doi: 10.1097/nt.0000000000000234

[8] Birch LL, Doub AE. Learning to eat: birth to age 2 y. Am J Clin Nutr. 2014;99: 723S–728S. doi: 10.3945/ajcn.113.069047 24452235

[9] Thompson AL, Mendez MA, Borja JB, Adair LS, Zimmer CR, Bentley ME. Development and validation of the Infant Feeding Style Questionnaire. Appetite. 2009;53: 210–221. doi: 10.1016/j.appet.2009.06.010 19576254 PMC3130353

[10] Khalsa AS, Woo JG, Kharofa RY, Geraghty SR, DeWitt TG, Copeland KA. Parental intuitive eating behaviors and their association with infant feeding styles among low-income families. Eat Behav. 2019;32: 78–84. doi: 10.1016/j.eatbeh.2019.01.001 30658288

[11] Spill MK, Callahan EH, Shapiro MJ, Spahn JM, Wong YP, Benjamin-Neelon SE, et al. Caregiver feeding practices and child weight outcomes: A systematic review. Am J Clin Nutr. 2019;109: 990S–1002S. doi: 10.1093/ajcn/nqy276 30982865

[12] Wood CT, Perreira KM, Perrin EM, Yin HS, Rothman RL, Sanders LM, et al. Confirmatory Factor Analysis of the Infant Feeding Styles Questionnaire in Latino Families Charles. 2017;100: 118–125. doi: 10.1016/j.appet.2016.02.018 26876910 PMC4799737

[13] Zhang X, Benton L. The association of acculturation and complementary infant and young child feeding practices among new chinese immigrant mothers in England: A mixed methods study. Int J Environ Res Public Health. 2019;16: 3282. doi: 10.3390/ijerph16183282 31500137 PMC6765794

[14] Van Der Veek SMC, De Graaf C, De Vries JHM, Jager G, Vereijken CMJL, Weenen H, et al. Baby’s first bites: A randomized controlled trial to assess the effects of vegetable-exposure and sensitive feeding on vegetable acceptance, eating behavior and weight gain in infants and toddlers. BMC Pediatr. 2019;19: 266. doi: 10.1186/s12887-019-1627-z 31370830 PMC6670176

[15] Barrett KJ, Wasser HM, Thompson AL, Bentley ME. Contributions of nonmaternal caregivers to infant feeding in a low-income African-American sample. Matern Child Nutr. 2018;14: e12610. doi: 10.1111/mcn.12610 29693776 PMC6156936

[16] Dancel LD, Perrin E, Yin SH, Sanders L, Delamater A, Perreira KM, et al. The Relationship Between Acculturation and Infant Feeding Styles in a Latino Population. Obesity. 2015;23: 840–846. doi: 10.1002/oby.20986 25755135 PMC4380799

[17] DiSantis KI, Hodges EA, Fisher JO. The association of breastfeeding duration with later maternal feeding styles in infancy and toddlerhood: A cross-sectional analysis. Int J Behav Nutr Phys Act. 2013;10: 53. doi: 10.1186/1479-5868-10-53 23621981 PMC3648372

[18] Doub AE. Infant and maternal predictors of early life feeding decisions: The timing of solid food introduction. Appetite. 2015;92: 261–268. doi: 10.1016/j.appet.2015.05.028 26025089 PMC4499500

[19] Messito MJ, Katzow MW, Mendelsohn AL, Gross RS. AFRI_Starting Early Program Impacts on Feeding at Infant 10 Months Age: A Randomized Controlled Trial. Child Obes. 2020. doi: 10.1089/chi.2019.0236 31934788 PMC7469695

[20] Pedroso J, Toral N, Gubert MB. Maternal attitudes, beliefs and practices related to the feeding and nutritional status of schoolchildren. Rev Nutr. 2019;32: e180184. doi: 10.1590/1678-9865201932e180184

[21] Lorenzato L, Cruz ISM, Costa TMB, Almeida SS. Translation and cross-cultural adaptation of a Brazilian version of the child feeding questionnaire. Paideia. 2017;27: 33–42. doi: 10.1590/1982-43272766201705

[22] Jellmayer K, Ganen AP, Alvarenga M. Influence of behavior and maternal perception on their children’s eating and nutritional status. O Mundo da Saúde. 2017;41: 180–193. doi: 10.15343/0104-7809.20174102180193

[23] Beaton DE, Bombardier C, Guillemin F, Ferraz MB. Guidelines for the Process of Cross-Cultural Adaptation of Self-Report Measures. Spine (Phila Pa 1976). 2000;25: 3186–3191. doi: 10.1097/00007632-200012150-00014 11124735

[24] Terwee CB, Bot SDM, de Boer MR, van der Windt DAWM, Knol DL, Dekker J, et al. Quality criteria were proposed for measurement properties of health status questionnaires. J Clin Epidemiol. 2007;60: 34–42. doi: 10.1016/j.jclinepi.2006.03.012 17161752

[25] Hair JF Jr, Anderson RE, Tatham RL, Black WC. Análise multivariada de dados. 5th ed. Porto Alegre: Bookman; 2005.

[26] Schreiber JB, Stage FK, King J, Nora A, Barlow EA. Reporting structural equation modeling and confirmatory factor analysis results: A review. J Educ Res. 2006;99: 323–338. doi: 10.3200/JOER.99.6.323–338

[27] Kline RB. Principles and Practice of Structural Equation Modeling. 4. ed. New York: Gilford Press; 2015.

[28] Instituto Brasileiro de Geografia e Estatística. Pesquisa Nacional de Saúde do Escolar. Rio de Janeiro; 2016.

[29] Ministério da Saúde. Orientações para avaliação de marcadores de consumo alimentar na atenção básica. Brasília; 2015.

[30] Thompson AL, Adair LS, Bentley ME. Pressuring and restrictive feeding styles influence infant feeding and size among a low-income African-American sample. Obesity. 2013;21: 562–571. doi: 10.1002/oby.20091 23592664 PMC3630475

[31] Garin O.Ceiling Effect. In: Michalos AC, editor. Encyclopedia of Quality of Life and Well-Being Research. Dordrecht: Springer; 2014. doi: 10.1007/978-94-007-0753-5_296

[32] Strainer DL, Norman GR, Cairney J. Health Measurement Scales: A practical guide to their development and use. 5. ed. Oxford: Oxford University Press; 2015.

[33] Rodrigues SLL, Rodrigues RCM, São-João TM, Pavan RBB, Padilha KM, Gallani MC. Impact of the disease: acceptability, ceiling and floor effects and reliability of an instrument on heart failure. Revista da Escola de Enfermagem da USP. 2013;47(5):1090–7. doi: 10.1590/S0080-623420130000500012 24346448

