# Supplementary material for: Cross-cultural adaptation and validation of the Infant Feeding Style Questionnaire in Brazil
Source: PLoS One. 2021 Sep 30;16(9):e0257991. doi: 10.1371/journal.pone.0257991 (PMC8483293; doi:10.1371/journal.pone.0257991)
Supplement: S1 Table — Federal District, Brazil. 2018. (DOCX) [file pone.0257991.s001.docx]

| S1 Table. Results of the steps for the cross-cultural adaptation of the Infant Feeding Style Questionnaire (IFSQ-Br). Federal District, Brazil. 2018. | | | | | | | | |
| --- | --- | --- | --- | --- | --- | --- | --- | --- |
| Item ^a^ | **Translation 1 (T1)** | **Translation 2 (T2)** | **Consolidated version** | **Back-translation 1 (BT1)** | **Back-translation 1 (BT2)** | **Pre-test version** | **IFSQ-Br** |  |
| Feeding style: Laissez-Faire | **Estilo de alimentação: Laissez-Faire** | **Estilo de alimentação: Laissez-Faire (por conta própria)** | **Estilo de alimentação: Laissez-Faire** | **Feeding Style: Laissez-Faire** | **Feeding Style: Laissez-Faire** | **Estilo de alimentação: Laissez-Faire** | **Estilo de alimentação: Laissez-Faire** |  |
| Attention | **Atenção** | **Atenção** | **Atenção** | **Attention** | **Attention** | **Atenção** | **Atenção** |  |
| LF1 | Quando (nome da criança) toma/tomava mamadeira, eu a seguro/segurava para cima | Quando (nome da criança) tem/tinha uma mamadeira, eu seguro/segurava | Quando (nome da criança) toma/tomava mamadeira, eu seguro/segurava (a mamadeira) para cima | When (child's name) takes/used to take the bottle I hold/used to hold (the bottle) up | When (child’s name) drinks/use to drink from a baby bottle, I hold/held (his/her) bottle up | Quando o/a (nome da criança) toma/tomava mamadeira, eu seguro/segurava (a mamadeira) para cima | Quando o/a (nome da criança) toma/tomava mamadeira, eu seguro/segurava (a mamadeira) para cima |  |
| LF2 | (A criança) assiste à TV durante a alimentação | (A criança) assiste TV enquanto come | (A criança) assiste TV enquanto come | (The child) watches TV while eating | (The child) watches TV while he/she eats | O/A (nome da criança) assiste TV enquanto come | O/A (nome da criança) assiste TV enquanto come |  |
| LF3 | Assisto à TV enquanto alimento (a criança) | Assisto TV enquanto alimento (a criança) | Eu assisto TV enquanto alimento (a criança) | I watch TV while feeding (the child) | I watch TV while I feed (the child) | Eu assisto TV enquanto dou comida para o/a (nome da criança) | Eu assisto TV enquanto dou comida para o/a (nome da criança) |  |
| LF4 | Não vejo problema em segurar a mamadeira do bebê | Acho que tudo bem segurar a mamadeira do bebê | Não vejo problema em segurar a mamadeira do bebê | I don't see any problem with holding the baby's bottle | I don’t see a problem in holding the baby’s bottle | Eu não vejo problema em alguém segurar a mamadeira para os bebês (enquanto eles estão mamando) | Eu não vejo problema em segurar a mamadeira para os bebês (enquanto eles estão mamando) |  |
| LF5 | Não tem problema crianças pequenas ficarem andando durante a alimentação, desde que comam | Não há problema deixar uma criança pequena (18 meses a 3 anos) andar enquanto come desde que ela esteja comendo | Não tem problema deixar uma criança pequena andar enquanto come, desde que ela coma | There's no problem if a small child eats while walking, as long as s/he eats | There is no problem in letting toddlers walk while they eat, as long as they do eat | Não tem problema deixar crianças pequenas ficarem andando enquanto comem, desde que elas comam | Não tem problema deixar crianças pequenas ficarem andando enquanto comem, desde que elas comam |  |
| Diet quality | **Qualidade da dieta** | **Diet quality** | **Qualidade da dieta** | **Diet Quality** | **Diet Quality** | **Qualidade da dieta** | **Qualidade da dieta** |  |
| LF6 | Controlo o que (a criança) come | Presto atenção na comida que (a criança) come | Controlo o que (a criança) come | I monitor what (the child) eats | I control what (the child) eats | Eu monitoro o que o/a (nome da criança) come | Eu monitoro o que o/a (nome da criança) come |  |
| LF7 | Controlo a quantidade de comida que (a criança) come | Presto atenção na quantidade que (a criança) come | Controlo a quantidade de comida que (a criança) come | I monitor the amount of food (the child) eats | I control the amount of food (the child) eats | Eu monitoro a quantidade de comida que o/a (nome da criança) come | Eu monitoro a quantidade de comida que o/a (nome da criança) come |  |
| LF8 | Eu me certifico para que (a criança) não coma alimentos açucarados, como bala, sorvete, bolo ou biscoito | Procuro evitar que (a criança) coma alimentos açucarados como doces, sorvetes, bolos ou biscoitos | Eu me certifico para que (a criança) não coma alimentos açucarados, como bala, sorvete, bolo ou biscoito | I ensure that the (child) does not eat sugary foods such as sweets, ice cream, cake or cookies | I make sure that (the child) doesn’t eat sugary foods, such as candy, ice cream, cakes or cookies | Eu fico atenta para que o/a (nome da criança) não coma alimentos açucarados, como balas, sorvetes, bolos ou biscoitos | Eu fico atenta para que o/a (nome da criança) não coma alimentos açucarados, como balas, sorvetes, bolos ou biscoitos |  |
| LF9 | Eu me certifico para que (a criança) não coma porcarias, como batatas chips, Doritos e salgadinhos de queijo | Procuro evitar que (a criança) coma “besteira” (junk food), tais como batata chips, Doritos e salgadinhos | Eu me certifico para que (a criança) não coma "besteiras", como batatas chips, Doritos e salgadinhos de queijo | I ensure that (the child) does not eat "junk food" such as potato chips, Doritos, and cheesy snacks | I make sure that (the child) doesn’t eat junk food, such as chips and Doritos | Eu fico atenta para que o/a (nome da criança) não coma "besteiras" ou "porcarias", como batatas fritas de pacote ou outros salgadinhos de pacote | Eu fico atenta para que o/a (nome da criança) não coma "besteiras" ou "porcarias", como batatas fritas de pacote ou outros salgadinhos de pacote |  |
| LF10 | As crianças pequenas devem poder comer o que quiserem no lanche | Uma criança pequena deveria comer o que quiser como guloseima | Uma criança pequena deve poder comer o que quiser nos lanches | A small child should be able to eat what s/he wants for snacks | Toddlers should be able to eat whatever they want for snack | Crianças pequenas deveriam poder comer o que quiserem nos lanches | Crianças pequenas deveriam poder comer o que quiserem nos lanches |  |
| LF11 | As crianças pequenas devem poder comer o que quiserem no restaurante | Uma criança pequena deveria comer o que quiser em um restaurante | Uma criança pequena deve poder comer o que quiser quando estiver comendo fora em um restaurante | A small child should be able to eat what s/he wants when eating out in a restaurant | Toddlers should be able to eat whatever they want when eating out at restaurants | Crianças pequenas deveriam poder comer o que quiserem quando estiverem comendo na rua ou fora de casa | Crianças pequenas deveriam poder comer o que quiserem quando estiverem comendo na rua ou fora de casa |  |
| Feeding style: Pressuring | **Estilo de alimentação: Controlador** | **Estilo de alimentação: Pressionando** | **Estilo de alimentação: Pressionador** | **Feeding style: Pressurizing** | **Feeding Style: Pressuring** | **Estilo de alimentação: Pressionador** | **Estilo de alimentação: Pressionador** |  |
| Finish | **Comer tudo** | **Término** | **Terminando** | **Finishing** | **Finishing** | **Terminando** | **Término** |  |
| PR1 | Tento fazer com que (a criança) coma toda a comida | Tento fazer com que (a criança) termine de comer o que foi servido | Tento fazer com que (a criança) termine de comer toda a sua comida | I try to make sure (the child) finishes eating all his/her food | I try to make (the child) finish all his/her food | Eu tento fazer com que o/a (nome da criança) termine de comer toda a sua comida | Eu tento fazer com que o/a (nome da criança) termine de comer toda a sua comida |  |
| PR2 | Se (a criança) parecer satisfeita, incentivo a comer tudo mesmo assim | Caso (a criança) pareça “cheia”, encorajo ela a terminar mesmo assim | Se (a criança) parecer cheia, incentivo a comer tudo mesmo assim | If (the child) seems to be full up, I still encourage him/her to eat everything | If (the child) seems satisfied, I encourage him/her to eat everything nonetheless | Mesmo se o/a (nome da criança) parecer cheio (a), incentivo a comer tudo mesmo assim | Mesmo se o/a (nome da criança) parecer cheio (a), incentivo a comer tudo mesmo assim |  |
| PR3 | Tento fazer com que (a criança) tome todo o leite materno ou mamadeira | Tento fazer com que (a criança) termine de tomar o leite materno ou fórmula pronta | Tento fazer com que (a criança) termine de tomar todo o leite materno ou mamadeira (fórmula) | I try to make sure (the child) finishes drinking all the breast milk or formula in the bottle | I try to make (the child) finish drinking all his/her breastmilk or formula | Eu tento fazer com que o/a (nome da criança) termine a mamada ou o leite da mamadeira (fórmula) | Eu tento fazer com que o/a (nome da criança) termine a mamada do peito ou o leite da mamadeira (fórmula) |  |
| PR4 | Tento fazer com que (a criança) coma mesmo sem fome | Tento fazer com que (a criança) coma mesmo sem fome | Tento fazer com que (a criança) coma mesmo sem fome | I try to make (the child) eat, even if s/he is not hungry | I try to make (the child) eat even if he/she is not hungry | Eu tento fazer com que o/a (nome da criança) coma mesmo se ele/ela estiver sem fome | Eu tento fazer com que o/a (nome da criança) coma mesmo se ele/ela estiver sem fome |  |
| PR5 | Insisto novamente alimentos desconhecidos que tenham sido recusados na mesma refeição | Insisto/tento novamente servir um novo alimento que foi recusado na mesma refeição | Insisto/tento novamente servir um novo alimento que foi recusado na mesma refeição | I persist/try again to serve a new food that has been refused at the same meal | I insist/try to serve again a new food that was rejected during that same meal | Eu insisto/tento novamente servir um novo alimento que o/a (nome da criança) não quis na mesma refeição | Eu insisto/tento novamente  servir um alimento novo que o/a (nome da criança) não quis naquela mesma refeição |  |
| PR6 | Elogio a cada colherada para incentivar a comer tudo | Parabenizo cada mordida para encorajar (a criança) a terminar de comer | Elogio a cada colherada para incentivar a comer tudo | I praise every spoonful to encourage him/her to eat everything | I praise each spoonful to encourage him/her to eat everything | Eu elogio a cada colherada para incentivar o/a (nome da criança) a comer tudo | Eu elogio a cada colherada para incentivar o/a (nome da criança) a comer tudo |  |
| PR7 | É importante que as crianças pequenas comam toda a comida no prato | É importante que uma criança pequena coma tudo que está no prato | É importante que uma criança pequena coma tudo que está no prato | It is important that a small child eats everything on his/her plate | It is important that toddlers eat everything that is on their plates | É importante que crianças pequenas comam tudo que está no prato | É importante que crianças pequenas comam tudo que está no prato |  |
| PR8 | É importante que os bebês tomem todo o leite na mamadeira | É importante que um bebê termine de tomar todo o leite em sua mamadeira | É importante que um bebê termine de tomar todo o leite em sua mamadeira | It's important that a baby drinks all the milk in the bottle | It is important that babies finish drinking the whole milk/formula that is in their bottles | É importante que bebês terminem de tomar todo o leite da mamadeira | É importante que bebês terminem de tomar todo o leite da mamadeira |  |
| Cereal | **Cereais** | **Cereais Infantis** | **Cereais** | **Cereal** | **Cereals** | **Cereais** | **Cereais** |  |
| PR11 | Dou/dava (à criança) cereais na mamadeira | Dou/dei cereal para (a criança) na mamadeira | Dou/dava (à criança) cereais engrossantes na mamadeira | I give/used to give (the child) cereal in his/her bottle | I add/added cereals to (the child)’s bottle | Eu dou/dava ao/à (nome da criança) cereais engrossantes (como amido de milho e farinhas) na mamadeira | Eu dou/dava ao/à (nome da criança) cereais engrossantes (como amido de milho e farinhas) na mamadeira |  |
| PR12 | Dar cereais na mamadeira ajuda os bebês a dormirem a noite toda | Cereal na mamadeira ajuda o bebê a dormir a noite toda | Cereais engrossantes na mamadeira ajudam o bebê a dormir a noite toda | Cereal in the baby bottle helps the baby sleep through the night | Cereals in the bottle help babies sleep through the night | Cereais engrossantes (como amido de milho e farinhas) na mamadeira ajudam os bebês a dormirem a noite toda | Cereais engrossantes (como amido de milho e farinhas) na mamadeira ajudam os bebês a dormirem a noite toda |  |
| PR13 | É bom colocar cereal na mamadeira porque ajuda os bebês a se sentirem saciadas | Colocar cereal na mamadeira é bom pois ajuda o bebê a se sentir saciado | Colocar cereal na mamadeira é bom pois ajuda o bebê a se sentir cheio | Putting cereal in the bottle is good as it helps the baby feel full | Adding cereal to the bottle is good, since it helps babies feel full | Colocar cereais engrossantes (como amido de milho e farinhas) na mamadeira é bom pois ajuda os bebês a se sentirem cheios | Colocar cereais engrossantes (como amido de milho e farinhas) na mamadeira é bom pois ajuda os bebês a se sentirem cheios |  |
| PR14 | Crianças com menos de 6 meses precisam de mais do que a mamadeira ou o leite materno para se sentirem saciadas | Um bebê com menos de 6 meses precisa mais que fórmula pronta ou leite materno para estar saciado | Um bebê com menos de 6 meses precisa de mais que mamadeira (fórmula) ou leite materno para se sentir cheio | A baby aged under 6 months needs more that the bottle (formula) or breast milk to feel full | Babies under 6 months old need more than formula or breastmilk to feel full | Bebês com menos de 6 meses precisam de outros alimentos além do leite da mamadeira (fórmula) ou leite materno para se sentirem cheios | Bebês com menos de 6 meses precisam de outros alimentos além do leite materno ou leite da mamadeira (fórmula) para se sentirem cheios |  |
| PR15 | Crianças com menos de 6 meses precisam de mais do que a mamadeira ou o leite materno para dormirem a noite toda | Um bebê com menos de 6 meses precisa mais que fórmula pronta ou leite materno para dormir a noite toda | Um bebê com menos de 6 meses precisa de mais do que a mamadeira (fórmula) ou o leite materno para dormirem a noite toda | Children aged under 6 months need more than the bottle (formula) or breast milk to sleep through the night | Children under 6 months old need more than formula or breastmilk to sleep through the night | Bebês com menos de 6 meses precisam de outros alimentos além do leite da mamadeira (fórmula) ou o leite materno para dormirem a noite toda | Bebês com menos de 6 meses precisam de outros alimentos além do leite materno ou leite da mamadeira (fórmula) para dormirem a noite toda |  |
| Soothing | **Acalmar** | **Acalmar** | **Acalmando** | **Calming** | **Calming** | **Acalmando** | **Acalmando** |  |
| PR16 | Quando (a criança) chora, eu a alimento imediatamente | Quando (a criança) chora, eu imediatamente a alimento | Quando (a criança) chora, eu a alimento imediatamente | When (the child) cries, I feed him/her immediately | When (the child) cries, I feed him/her immediately | Quando o/a (nome da criança) chora, eu o/ a alimento imediatamente | Quando o/a (nome da criança) chora, eu o/ a alimento imediatamente |  |
| PR17 | A melhor maneira de fazer um bebê parar de chorar é alimentando-a | A melhor forma de fazer um bebê parar de chorar é alimentando-o | A melhor forma de fazer um bebê parar de chorar é alimentando-o | The best way of getting a baby to stop crying is to feed him/her | The best way to make babies stop crying is by feeding them | A melhor forma de fazer bebês pararem de chorar é alimentando-os | A melhor forma de fazer bebês pararem de chorar é alimentando-os |  |
| PR18 | A melhor maneira de fazer uma criança pequena parar de chorar é alimentando-a | A melhor forma de fazer uma criança pequena parar de chorar é alimentando-a | A melhor forma de fazer uma criança pequena parar de chorar é alimentando-a | The best way of getting a small child to stop crying is to feed him/her | The best way to make toddlers stop crying is by feeding them | A melhor forma de fazer crianças pequenas pararem de chorar é dando comida a elas | A melhor forma de fazer crianças pequenas pararem de chorar é dando comida a elas |  |
| PR19 | Quando os bebês choram, geralmente estão com fome | Quando o bebê chora, geralmente significa que ele precisa ser alimentado | Quando os bebês choram, geralmente significa que estão com fome | When babies cry, it usually means s/he is hungry | When babies cry, it usually means they are hungry | Quando os bebês choram, geralmente significa que estão com fome | Quando os bebês choram, geralmente significa que estão com fome |  |
| Feeding style: Restrictive | **Estilo de alimentação: Restritivo** | **Estilo de alimentação: Restritivo** | **Estilo de alimentação: Restritivo** | **Feeding style: Restrictive** | **Feeding Style: Restrictive** | **Estilo de alimentação: Restritivo** | **Estilo de alimentação: Restritivo** |  |
| Amount | **Quantidade** | **Quantidades** | **Quantidade** | **Quantity** | **Quantity** | **Quantidade** | **Quantidade** |  |
| RS1 | Controlo com cuidado a quantidade de comida que (a criança) come | Controlo cuidadosamente o quanto (a criança) come | Eu controlo com cuidado a quantidade de comida que (a criança) come | I carefully monitor the amount of food (the child) eats | I control carefully the amount of food (the child) eats | Eu controlo com cuidado a quantidade de comida que o/a (nome da criança) come | Eu controlo com cuidado a quantidade de comida que o/a (nome da criança) come |  |
| RS2 | Tomo bastante cuidado para não alimentar (a criança) em excesso | Tomo cuidado para não alimentar (a criança) além do necessário | Eu tomo bastante cuidado para não alimentar (a criança) em excesso | I am careful not to overfeed (the child) | I am very careful not to overfeed (the child) | Eu tomo bastante cuidado para não alimentar o/a (nome da criança) demais/em excesso | Eu tomo bastante cuidado para não alimentar o/a (nome da criança) demais/em excesso |  |
| RS3 | É importante que os pais tenham regras sobre a quantidade de comida que as crianças pequenas comem | É importante que os pais tenham regras sobre o quanto as crianças pequenas comem | É importante que os pais tenham regras sobre a quantidade de comida que as crianças pequenas comem | It is important that parents set rules about the amount of food small children eat | It is important that parents have rules regarding the amount of food toddlers eat | É importante que os pais tenham regras sobre a quantidade de comida que as crianças pequenas devem comer | É importante que os pais tenham regras sobre a quantidade de comida que as crianças pequenas devem comer |  |
| RS4 | É importante que os pais definam a quantidade de comida que os bebês devem comer | É importante que os pais decidam quanto um bebê deve comer | É importante que os pais decidam a quantidade de comida que os bebês devem comer | It is important that the parents decide how much food the babies should eat | It is important that parents decide the amount of food babies should eat | É importante que os pais decidam a quantidade de comida que os bebês devem comer | É importante que os pais decidam a quantidade de comida que os bebês devem comer |  |
| Diet quality | **Qualidade da dieta** | **Qualidade da alimentação** | **Qualidade da alimentação** | **Food quality** | **Food Quality** | **Qualidade da alimentação** | **Qualidade da alimentação** |  |
| RS5 | Deixo (a criança) comer fast food | Deixo (a criança) consumir fast food | Deixo (a criança) comer fast food | I let (the child) eat fast food | I let (the child) eat fast food | Eu deixo o/a (nome da criança) comer fast food (comida pronta rápida servida em lanchonetes, barracas de cachorro quentes, pizzaria...). | Eu deixo o/a (nome da criança) comer fast food (comida pronta rápida servida em lanchonetes, barracas de cachorro quentes, pizzaria...). |  |
| RS6 | Deixo (a criança) comer porcarias | Deixo (a criança) consumir “besteira” (junk food) | Deixo (a criança) comer “besteira” | I let (the child) eat “junk food” | I let (the child) eat junk food | Eu deixo o/a (nome da criança) comer “besteiras” ou "porcarias" (como salgadinho de pacote, balinhas, biscoitos recheados...) | Eu deixo o/a (nome da criança) comer “besteiras” ou "porcarias" (como salgadinho de pacote, balinhas, biscoitos recheados...) |  |
| RS7 | As crianças pequenas nunca devem comer fast-food | Uma criança pequena nunca deve comer fast food | Uma criança pequena nunca deve comer fast food | A small child shouldn't eat fast food | Toddlers should never eat fast food | Crianças pequenas nunca devem comer fast food (comida pronta rápida servida em lanchonetes, barracas de cachorro quentes, pizzaria...) | Crianças pequenas nunca devem comer fast food (comida pronta rápida servida em lanchonetes, barracas de cachorro quentes, pizzaria...) |  |
| RS8 | Os bebês nunca devem comer fast-food | Um bebê nunca deve comer fast food | Um bebê nunca deve comer fast food | A baby should never eat fast food | Babies should never eat fast food | Bebês nunca devem comer fast food (comida pronta rápida servida em lanchonetes, barracas de cachorro quentes, pizzaria...) | Bebês nunca devem comer fast food (comida pronta rápida servida em lanchonetes, barracas de cachorro quentes, pizzaria...) |  |
| RS9 | As crianças pequenas nunca devem comer alimentos açucarados, como biscoitos | Uma criança pequena nunca deve comer alimentos açucarados, tais como biscoitos | Uma criança pequena nunca deve comer alimentos açucarados, como biscoitos | A small child should never eat sugary food such as cookies | Toddlers should never eat sugary foods, such as cookies | Crianças pequenas nunca devem comer alimentos açucarados, como biscoitos recheados | Crianças pequenas nunca devem comer alimentos açucarados, como biscoitos recheados |  |
| RS10 | As crianças pequenas nunca devem comer porcarias, como batatas chips | Uma criança pequena nunca deve comer “besteiras” (junk food), tais como batata chips | Uma criança pequena nunca deve comer “besteiras”, como batata chips | A small child should never eat "junk food" such as potato chips | Toddlers should never eat junk food, such as chips | Crianças pequenas nunca devem comer “besteiras” ou "porcarias", como salgadinhos de pacote | Crianças pequenas nunca devem comer “besteiras” ou "porcarias", como salgadinhos de pacote |  |
| RS11 | As crianças pequenas só devem comer alimentos saudáveis | Uma criança pequena deveria consumir apenas comida saudável | Uma criança pequena deve comer apenas alimentos saudáveis | A small child should only eat healthy food | Toddlers should only eat healthy foods | Crianças pequenas devem comer apenas alimentos e comidas saudáveis | Crianças pequenas devem comer apenas alimentos e comidas saudáveis |  |
| Feeding style: Responsive | **Estilo de alimentação: Responsivo** | **Estilo de alimentação: Responsiva** | **Estilo de alimentação: Responsivo** | **Feeding style: Responsive** | **Feeding Style: Responsive** | **Estilo de alimentação: Responsivo** | **Estilo de alimentação: Responsivo** |  |
| Satiety | **Saciedade** | **Saciedade** | **Saciedade** | **Satiety** | **Fullness** | **Saciedade** | **Saciedade** |  |
| RP1 | (A criança) me diz quando está satisfeita | (A criança) me diz quando está saciada | A criança me deixa saber quando ela está cheia | The child lets me know when s/he is full up | My child lets me know when he/she is full | O/A (nome da criança) me mostra quando ele/ela está cheio (a) | O/A (nome da criança) me mostra quando ele/ela está cheio (a) |  |
| RP2 | (A criança) me diz quando está com fome | (A criança) me diz quando está com fome | A criança me deixa saber quando ela está com fome | The child lets me know when s/he is hungry | My child lets me know when he/she is hungry | O/A (nome da criança) me mostra quando ela/ele está com fome | O/A (nome da criança) me mostra quando ela/ele está com fome |  |
| RP3 | Deixo (a criança) decidir o quanto comer | Deixo (a criança) decidir a quantidade que vai comer | Eu deixo (a criança) decidir a quantidade que vai comer | I let (the child) decide how much s/he wants to eat | I let (the child) decide the amount of food he/she is eating | Eu deixo o/a (nome da criança) decidir o quanto vai comer | Eu deixo o/a (nome da criança) decidir o quanto vai comer |  |
| RP4 | Presto atenção quando (a criança) dá indícios de estar satisfeita ou com fome | Presto atenção quando (a criança) parece me dizer que está saciada ou com fome | Eu presto atenção quando (a criança) parece me dizer que ela está cheia ou com fome | I pay attention when (the child) seems to be telling me s/he is full or is hungry | I pay attention when (the child) seems to tell me that he/she is full or hungry | Eu presto atenção quando o/a (nome da criança) parece me dizer que ele/ela está cheio (a) ou com fome | Eu presto atenção quando o/a (nome da criança) parece me dizer que ele/ela está cheio (a) ou com fome |  |
| RP5 | Permito que (a criança) coma quando está com fome | Deixo (a criança) comer quando está com fome | Eu permito que (a criança) coma quando ela está com fome | I allow (the child) to eat when s/he is hungry | I let (the child) eat when he/she is hungry | Eu permito que o/a (nome da criança) coma quando ele/ela está com fome | Eu permito que o/a (nome da criança) coma quando ele/ela está com fome |  |
| RP6 | A criança sabe quando está satisfeita | A criança sabe quando está saciada | A criança sabe quando ela está cheia | The child knows when s/he is hungry | Children know when they are full | Crianças sabem quando elas estão cheias | As crianças sabem quando elas estão cheias |  |
| RP7 | A criança sabe quando está com fome, precisa comer | A criança sabe quando está com fome e precisa comer | A criança sabe quando está com fome e precisa comer | The child knows when s/he is hungry and needs to eat | Children know when they are hungry and need to eat | Crianças sabem quando estão com fome e precisam comer | As crianças sabem quando estão com fome e precisam comer |  |
| Attention | **Atenção** | **Atenção** | **Atenção** | **Attention** | **Attention** | **Atenção** | **Atenção** |  |
| RP8 | Converso (com a criança) para incentivá-la a tomar a mamadeira/o leite materno | Falo com (a criança) para encorajá-la a beber fórmula pronta/leite materno | Converso (com a criança) para incentivá-la a tomar mamadeira (fórmula)/leite materno | I talk (with the child) to encourage him/her to take the bottle (formula)/breast milk | I talk (to the child) to encourage him/her to drink his/her formula/breast milk | Eu converso com o/a (nome da criança) para incentivá-lo (a) a tomar mamadeira (fórmula)/ou mamar no peito | Eu converso com o/a (nome da criança) para incentivá-lo (a) a tomar mamadeira (fórmula)/ou mamar no peito |  |
| RP9 | Converso (com a criança) para incentivá-la a comer | Falo com (a criança) para encorajá-la a comer | Converso (com a criança) para incentivá-la a comer | I talk (with the child) to encourage him/her to eat | I talk (to the child) to encourage him/her to eat | Eu converso com o/a (nome da criança) para incentivá-lo (a) a comer | Eu converso com o/a (nome da criança) para incentivá-lo (a) a comer |  |
| RP10 | Mostro (à criança) como comer comendo uma colherada ou fingindo | Mostro para (a criança) como comer dando uma mordida ou fingindo dar uma mordida | Mostro para (a criança) como comer dando uma mordida ou fingindo dar uma mordida | I show (the child) how to eat, by taking, or pretending to take, a bite | I show (the child) how to eat by taking a bite or pretending to take a bite | Eu mostro para o/a (nome da criança) como comer dando uma mordida ou fingindo morder | Eu mostro para o/a (nome da criança) como comer dando uma mordida ou fingindo morder |  |
| RP11 | Quando alimentos desconhecidos são recusados pela primeira vez tento dá-los novamente | Vou tentar introduzir novos alimentos caso sejam recusados na primeira vez | Quando alimentos desconhecidos são recusados pela primeira vez tento dá-los novamente | When unfamiliar food is refused for the first time, I try to give it again | When unknown foods are rejected the first time, I try to offer them again | Eu tento oferecer novamente ao/à (nome da criança) alimentos que são rejeitados na primeira vez | Eu tento oferecer novamente ao/à (nome da criança) alimentos que ele /ela rejeitou quando ofereci na primeira vez |  |
| RP12 | É importante ajudar ou incentivar as crianças pequenas a comerem | É importante ajudar ou encorajar uma criança pequena a comer | É importante ajudar ou incentivar uma criança pequena a comer | It is important to help or encourage a small child to eat | It is important to help or encourage toddlers to eat | É importante ajudar ou incentivar crianças pequenas a comerem | É importante ajudar ou incentivar crianças pequenas a comerem |  |
| Feeding style: Indulgence | **Estilo de alimentação: Indulgente** | **Estilo de alimentação: Indulgente** | **Estilo de alimentação: Indulgente** | **Feeding style: Indulgent** | **Feeding Style: Indulging** | **Estilo de alimentação: Indulgente** | **Estilo de alimentação: Indulgente** |  |
| Permissive | **Permissivo** | **Permissividade** | **Permissivo** | **Permissive** | **Permissive** | **Permissivo** | **Permissivo** |  |
| ID1 | Permito que a criança assista à TV durante a alimentação, se quiser | Deixo que a criança assista TV enquanto come caso ela queira | Permito que a criança assista TV enquanto come caso ela queira | I allow the child to watch TV while eating if s/he wants to | I let my child watch TV while he/she eats if he/she wants to | Eu permito que o/a (nome da criança) assista TV enquanto come caso ele/ela queira | Eu permito que o/a (nome da criança) assista TV enquanto come caso ele/ela queira |  |
| ID2 | Permito que a criança coma fast food, se quiser | Deixo que a criança coma fast food caso ela queira | Permito que a criança coma fast food caso ela queira | I allow the child to eat fast food if s/he wants to | I let my child eat fast food if he/she wants to | Eu permito que o/a (nome da criança) coma fast food (comida pronta rápida servida em lanchonetes, barracas de cachorro quentes, pizzaria...) caso ele/ela queira | Eu permito que o/a (nome da criança) coma fast food (comida pronta rápida servida em lanchonetes, barracas de cachorro quentes, pizzaria...) caso ele/ela queira |  |
| ID3 | Permito que a criança beba bebidas açucaradas/ refrigerantes, se quiser | Deixo que a criança beba refrigerantes/bebidas açucaras caso ela queira | Permito que a criança beba bebidas açucaradas/refrigerantes caso ela queira | I allow the child to drink sugary drinks/soda if s/he wants to | I let my child drink sugary/soft drinks if he/she wants to | Eu permito que o/a (nome da criança) tome bebidas açucaradas (como suco de caixinha, suco em pó, água de coco de caixinha, xaropes de guaraná/groselha, suco de fruta com adição de açúcar)/refrigerantes caso ele/ela queira | Eu permito que o/a (nome da criança) tome bebidas açucaradas (como suco de caixinha, suco em pó, água de coco de caixinha, xaropes de guaraná/groselha, suco de fruta com adição de açúcar)/refrigerantes caso ele/ela queira |  |
| ID4 | Permito que a criança coma sobremesas/doces, se quiser | Deixo que a criança coma sobremesa/doces caso ela queira | Permito que a criança coma sobremesa/doces caso ela queira | I allow the child to eat dessert/sweets if s/he wants to | I let my child eat dessert/candy if he/she wants to | Eu permito que o/a (nome da criança) coma sobremesa/doces caso ele/ela queira | Eu permito que o/a (nome da criança) coma sobremesa/doces caso ele/ela queira |  |
| ID5 | As crianças pequenas devem ter permissão para assistir à TV durante a alimentação se quiserem | Crianças pequenas devem ter permissão de assistir TV enquanto comem caso queiram | Crianças pequenas deveriam poder assistir TV enquanto comem caso elas queiram | Small children should be able to watch TV while eating if they want to | Toddlers should be allowed to watch TV while they eat if they want to | Se as crianças pequenas quiserem, deve-se permitir que elas assistam TV enquanto comem. | Se as crianças pequenas quiserem, deve-se permitir que elas assistam TV enquanto comem. |  |
| ID6 | As crianças pequenas devem ter permissão para comer fast-food, se quiserem | Crianças pequenas devem ter permissão de comer fast food caso queiram | Crianças pequenas deveriam poder comer fast food caso elas queiram | Small children should be able to eat fast food if they want to | Toddlers should be allowed to eat fast food if they want to | Se as crianças pequenas quiserem, deve-se permitir que elas comam fast food (comida pronta rápida servida em lanchonetes, barracas de cachorro quentes, pizzaria...) | Se as crianças pequenas quiserem, deve-se permitir que elas comam fast food (comida pronta rápida servida em lanchonetes, barracas de cachorro quentes, pizzaria...) |  |
| ID7 | As crianças pequenas devem ter permissão para beber bebidas açucaradas/refrigerantes, se quiserem | Crianças pequenas devem ter permissão de beber refrigerantes/bebidas açucaradas caso queiram | Crianças pequenas deveriam poder beber refrigerantes/bebidas açucaradas caso elas queiram | Small children should be able to drink soda/sugary drinks if they want to | Toddlers should be allowed to drink soft/sugary drinks if they want to | Se as crianças pequenas quiserem, deve-se permitir que elas tomem bebidas açucaradas (como suco de caixinha, suco em pó, água de coco de caixinha, xaropes de guaraná/groselha, suco de fruta com adição de açúcar)/ refrigerantes. | Se as crianças pequenas quiserem, deve-se permitir que elas tomem bebidas açucaradas(como suco de caixinha, suco em pó, água de coco de caixinha, xaropes de guaraná/groselha, suco de fruta com adição de açúcar)/ refrigerantes. |  |
| ID8 | As crianças pequenas devem ter permissão para comer sobremesas/doces, se quiserem | Crianças pequenas devem ter permissão de comer sobremesas/doces caso queiram | Crianças pequenas deveriam poder comer sobremesas/doces caso elas queiram | Small children should be able to eat dessert/sweets if they want to | Toddlers should be allowed to eat dessert/candy if they want to | Se as crianças pequenas quiserem, deve-se permitir que elas comam sobremesas/doces. | Se as crianças pequenas quiserem, deve-se permitir que elas comam sobremesas/doces. |  |
| Coaxing | **Estímulos** | **Persuasão** | **Persuadindo** | **Persuasive** | **Persuading** | **Persuadindo** | **Persuadindo** |  |
| ID9 | Permito que a criança assista à TV durante a alimentação para garantir que coma o suficiente | Permito que a criança assista TV enquanto come para garantir que ela está saciada | Permito que a criança assista TV enquanto come para garantir que ela coma o suficiente | I allow the child to watch TV while eating to ensure s/he eats enough | I let my child watch TV while he/she eats to ensure that he/she eats enough | Eu permito que o/a (nome da criança) assista TV enquanto come para garantir que ele/ela coma o suficiente | Eu permito que o/a (nome da criança) assista TV enquanto come para garantir que ele/ela coma o suficiente |  |
| ID10 | Permito que a criança coma fast-food para garantir que coma o suficiente | Permito que a criança coma fast food para garantir que ela está saciada | Permito que a criança coma fast food para garantir que ela coma o suficiente | I allow the child to eat fast food to ensure s/he eats enough | I let my child eat fast food to ensure that he/she eats enough | Eu permito que o/a (nome da criança) coma fast food (comida pronta rápida servida em lanchonetes, barracas de cachorro quentes, pizzaria...) para garantir que ele/ela coma o suficiente | Eu permito que o/a (nome da criança) coma fast food (comida pronta rápida servida em lanchonetes, barracas de cachorro quentes, pizzaria...) para garantir que ele/ela coma o suficiente |  |
| ID11 | Permito que a criança beba bebidas açucaradas/ refrigerantes para garantir que beba o suficiente | Permito que a criança beba refrigerante/bebidas açucaradas para garantir que ela está saciada | Permito que a criança beba bebidas açucaradas/refrigerantes para garantir que ela beba o suficiente | I allow the child to drink sugary drinks/soda to ensure s/he drinks enough | I let my child drink sugary/soft drinks to ensure that he/she drinks enough | Eu permito que o/a (nome da criança) tome bebidas açucaradas (como suco de caixinha, suco em pó, água de coco de caixinha, xaropes de guaraná/groselha, suco de fruta com adição de açúcar)/refrigerantes para garantir que ele/ela beba o suficiente | Eu permito que o/a (nome da criança) tome bebidas açucaradas (como suco de caixinha, suco em pó, água de coco de caixinha, xaropes de guaraná/groselha, suco de fruta com adição de açúcar)/refrigerantes para garantir que ele/ela beba o suficiente |  |
| ID12 | Permito que a criança coma sobremesas/doces para garantir que coma o suficiente | Permito que a criança coma sobremesas/doces para garantir que ela está saciada | Permito que a criança coma sobremesas/doces para garantir que ela coma o suficiente | I allow the child to eat dessert/sweets to ensure s/he eats enough | I let my child eat dessert/candy to ensure that he/she eats enough | Eu permito que o/a (nome da criança) coma sobremesas/doces para garantir que ele/ela coma o suficiente | Eu permito que o/a (nome da criança) coma sobremesas/doces para garantir que ele/ela coma o suficiente |  |
| ID13 | As crianças pequenas devem ter permissão para assistir à TV durante a alimentação para garantir que se alimentem bem | Crianças pequenas devem ter permissão de assistir TV enquanto comem para garantirmos que estão saciadas | Crianças pequenas deveriam poder assistir TV enquanto comem para garantir que elas comam o suficiente | Small children should be able to watch TV while eating to ensure they eat enough | Toddlers should be allowed to watch TV while they eat to ensure that they eat enough | Para garantir que as crianças pequenas comam o suficiente, deve-se permitir que elas assistam TV enquanto comem. | Para garantir que as crianças pequenas comam o suficiente, deve-se permitir que elas assistam TV enquanto comem. |  |
| ID14 | As crianças pequenas devem ter permissão para comer fast-food para garantir que comam o suficiente | Crianças pequenas devem ter permissão de comer fast food para garantirmos que estão saciadas | As crianças pequenas deveriam poder comer fast-food para garantir que elas comam o suficiente | Small children should be able to eat fast food to ensure they eat enough | Toddlers should be allowed to eat fast food to ensure that they eat enough | Para garantir que as crianças pequenas comam o suficiente, deve-se permitir que elas comam fast-food (comida pronta rápida servida em lanchonetes, barracas de cachorro quentes, pizzaria...) | Para garantir que as crianças pequenas comam o suficiente, deve-se permitir que elas comam fast-food (comida pronta rápida servida em lanchonetes, barracas de cachorro quentes, pizzaria...) |  |
| ID15 | As crianças pequenas devem ter permissão para beber bebidas açucaradas/refrigerantes para garantir que bebam o suficiente | Crianças pequenas deveriam poder beber refrigerantes/bebidas açucaradas para garantir que estão saciadas | As crianças pequenas deveriam poder beber bebidas açucaradas/refrigerantes para garantir que elas bebam o suficiente | Small children should be able to drink sugary drinks/soda to ensure they drink enough | Toddlers should be allowed to drink sugary/soft drinks to ensure that they drink enough | Para garantir que as crianças pequenas bebam o suficiente, deve-se permitir que elas tomem bebidas açucaradas (como suco de caixinha, suco em pó, água de coco de caixinha, xaropes de guaraná/groselha, suco de fruta com adição de açúcar)/refrigerantes | Para garantir que as crianças pequenas bebam o suficiente, deve-se permitir que elas tomem bebidas açucaradas (como suco de caixinha, suco em pó, água de coco de caixinha, xaropes de guaraná/groselha, suco de fruta com adição de açúcar)/refrigerantes |  |
| ID16 | As crianças pequenas devem ter permissão para comer sobremesas/doces para garantir que comam o suficiente | Crianças pequenas deveriam poder comer sobremesas/doces para garantir que estão saciadas | As crianças pequenas deveriam poder comer sobremesas/doces para garantir que elas comam o suficiente | Small children should be able to eat dessert/sweet to ensure they eat enough | Toddlers should be allowed to eat dessert/candy to ensure that they eat enough | Para garantir que as crianças pequenas comam o suficiente, deve-se permitir que elas comam sobremesas/doces. | Para garantir que as crianças pequenas comam o suficiente, deve-se permitir que elas comam sobremesas/doces. |  |
| Soothing | **Acalmar** | **Acalmar** | **Acalmando** | **Calming** | **Calming** | **Acalmando** | **Acalmando** |  |
| ID17 | Permito que a criança assista à TV durante a alimentação para que não chore | Permito que a criança assista TV enquanto come para evitar que ela chore | Permito que a criança assista TV enquanto come para evitar que ela chore | I allow the child to watch TV while eating to avoid him/her crying | I let my child watch TV while he/she eats to avoid crying | Eu permito que o/a (nome da criança) assista TV enquanto come para evitar que ele/ela chore | Eu permito que o/a (nome da criança) assista TV enquanto come para evitar que ele/ela chore |  |
| ID18 | Permito que a criança coma fast food para que não chore | Permito que a criança coma fast food para evitar que ela chore | Permito que a criança coma fast food para evitar que ela chore | I allow the child to eat fast food to avoid him/her crying | I let my child eat fast food to avoid crying | Eu permito que o/a (nome da criança) coma fast food (comida pronta rápida servida em lanchonetes, barracas de cachorro quentes, pizzaria...) para evitar que ele/ela chore | Eu permito que o/a (nome da criança) coma fast food (comida pronta rápida servida em lanchonetes, barracas de cachorro quentes, pizzaria...) para evitar que ele/ela chore |  |
| ID19 | Permito que a criança beba bebidas açucaradas/ refrigerantes para que não chore | Permito que a criança beba refrigerantes/bebidas açucaradas para evitar que ela chore | Permito que a criança beba refrigerantes/bebidas açucaradas para evitar que ela chore | I allow the child to drink soda/sugary drinks to avoid him/her crying | I let my child drink soft/sugary drinks to avoid crying | Eu permito que o/a (nome da criança) tome bebidas açucaradas (como suco de caixinha, suco em pó, água de coco de caixinha, xaropes de guaraná/groselha, suco de fruta com adição de açúcar)/ refrigerantes para evitar que ele/ela chore | Eu permito que o/a (nome da criança) tome bebidas açucaradas (como suco de caixinha, suco em pó, água de coco de caixinha, xaropes de guaraná/groselha, suco de fruta com adição de açúcar)/ refrigerantes para evitar que ele/ela chore |  |
| ID20 | Permito que a criança coma sobremesas/doces para que não chore | Permito que a criança coma sobremesa/doces para evitar que ela chore | Permito que a criança coma sobremesa/doces para evitar que ela chore | I allow the child to eat dessert/sweets to avoid him/her crying | I let my child eat fast food to avoid crying | Eu permito que o/a (nome da criança) coma sobremesa/doces para evitar que ele/ela chore | Eu permito que o/a (nome da criança) coma sobremesa/doces para evitar que ele/ela chore |  |
| ID21 | As crianças pequenas devem ter permissão para assistir à TV durante a alimentação para que não chorem | Crianças pequenas devem ter permissão de assistir TV enquanto comem para evitar que elas chorem | Crianças pequenas deveriam poder assistir TV enquanto comem para evitar que elas chorem | Small children should be able to watch TV while eating to avoid them crying | Toddlers should be allowed to watch TV while they eat to avoid crying | Para evitar que crianças pequenas chorem, deve-se permitir que elas assistam TV enquanto comem. | Para evitar que crianças pequenas chorem, deve-se permitir que elas assistam TV enquanto comem. |  |
| ID22 | As crianças pequenas devem ter permissão para comer fast-food para que não chorem | Crianças pequenas devem ter permissão de comer fast food para evitar que elas chorem | Crianças pequenas deveriam poder comer fast food para evitar que elas chorem | Small children should be able to eat fast food to avoid them crying | Toddlers should be allowed to eat fast food to avoid crying | Para evitar que crianças pequenas chorem, deve-se permitir que elas comam fast food (comida pronta rápida servida em lanchonetes, barracas de cachorro quentes, pizzaria...) | Para evitar que crianças pequenas chorem, deve-se permitir que elas comam fast food (comida pronta rápida servida em lanchonetes, barracas de cachorro quentes, pizzaria...) |  |
| ID23 | As crianças pequenas devem ter permissão para beber bebidas açucaradas/refrigerantes para que não chorem | Crianças pequenas deveriam poder beber refrigerante/bebidas açucaradas para evitar que elas chorem | Crianças pequenas deveriam poder beber refrigerante/bebidas açucaradas para evitar que elas chorem | Small children should be able to drink soda/sugary drinks to avoid them crying | Toddlers should be allowed to drink soft/sugary drinks to avoid crying | Para evitar que crianças pequenas chorem, deve-se permitir que elas tomem bebidas açucaradas (como suco de caixinha, suco em pó, água de coco de caixinha, xaropes de guaraná/groselha, suco de fruta com adição de açúcar)/ refrigerantes. | Para evitar que crianças pequenas chorem, deve-se permitir que elas tomem bebidas açucaradas (como suco de caixinha, suco em pó, água de coco de caixinha, xaropes de guaraná/groselha, suco de fruta com adição de açúcar)/ refrigerantes. |  |
| ID24 | As crianças pequenas devem ter permissão para comer sobremesas/doces para que não chorem | Crianças pequenas deveriam poder comer sobremesas/doces para evitar que elas chorem | Crianças pequenas deveriam poder comer sobremesas/doces para evitar que elas chorem | Small children should be able to eat dessert/sweets to avoid them crying | Toddlers should be allowed to eat dessert/candy to avoid crying | Para evitar que crianças pequenas chorem, deve-se permitir que elas comam sobremesas/doces. | Para evitar que crianças pequenas chorem, deve-se permitir que elas comam sobremesas/doces. |  |
| Pampering | **Mimos** | **Mimando** | **Mimando** | **Spoiling** | **Spoiling** | **Mimando** | **Mimando** |  |
| ID25 | Permito que a criança assista à TV durante a alimentação para deixá-la feliz | Permito que a criança assista TV enquanto come para deixá-la feliz | Permito que a criança assista TV enquanto come para deixá-la feliz | I allow the child to watch TV while eating to keep him/her happy | I let my child watch TV while he/she eats to make him/her happy | Eu permito que o/a (nome da criança) assista TV enquanto come para deixá-lo (a) feliz | Eu permito que o/a (nome da criança) assista TV enquanto come para deixá-lo (a) feliz |  |
| ID26 | Permito que a criança coma fast food para deixá-la feliz | Permito que a criança coma fast food para deixá-la feliz | Permito que a criança coma fast food para deixá-la feliz | I allow the child to eat fast food to keep him/her happy | I let my child eat fast food to make him/her happy | Eu permito que o/a (nome da criança) coma fast food (comida pronta rápida servida em lanchonetes, barracas de cachorro quentes, pizzaria...) para deixá-lo (a) feliz | Eu permito que o/a (nome da criança) coma fast food (comida pronta rápida servida em lanchonetes, barracas de cachorro quentes, pizzaria...) para deixá-lo (a) feliz |  |
| ID27 | Permito que a criança beba bebidas açucaradas/refrigerantes para deixá-la feliz | Permito que a criança beba refrigerantes/bebidas açucaradas para deixá-la feliz | Permito que a criança beba refrigerantes/bebidas açucaradas para deixá-la feliz | I allow the child to drink soda/sugary drinks to keep him/her happy | I let my child drink soft/sugary drinks to make him/her happy | Eu permito que o/a (nome da criança) tome bebidas açucaradas (como suco de caixinha, suco em pó, água de coco de caixinha, xaropes de guaraná/groselha, suco de fruta com adição de açúcar)/ refrigerantes para deixá-lo (a) feliz | Eu permito que o/a (nome da criança) tome bebidas açucaradas (como suco de caixinha, suco em pó, água de coco de caixinha, xaropes de guaraná/groselha, suco de fruta com adição de açúcar)/ refrigerantes para deixá-lo (a) feliz |  |
| ID28 | Permito que a criança coma sobremesas/doces para deixá la feliz | Permito que a criança coma sobremesa/doces para deixá-la feliz | Permito que a criança coma sobremesa/doces para deixá-la feliz | I allow the child to eat dessert/sweets to keep him/her happy | I let my child eat dessert/candy to make him/her happy | Eu permito que o/a (nome da criança) coma sobremesa/doces para deixá-lo (a) feliz | Eu permito que o/a (nome da criança) coma sobremesa/doces para deixá-lo (a) feliz |  |
| ID29 | As crianças pequenas devem ter permissão para assistir à TV durante a alimentação, para que fiquem felizes | Crianças pequenas devem ter permissão de assistir TV enquanto comem para que fiquem felizes | Crianças pequenas deveriam poder assistir TV enquanto comem para que fiquem felizes | Small children should be able to watch TV while eating to keep them happy | Toddlers should be allowed to watch TV while they eat to make them happy | Para que as crianças pequenas fiquem felizes, deve-se deve-se permitir que elas assistam TV enquanto comem. | Para que as crianças pequenas fiquem felizes, deve-se deve-se permitir que elas assistam TV enquanto comem. |  |
| ID30 | As crianças pequenas devem ter permissão para comer fast-food, para que fiquem felizes | Crianças pequenas devem ter permissão de comer fast food para que fiquem felizes | Crianças pequenas deveriam poder comer fast food para que fiquem felizes | Small children should be able to eat fast food to keep them happy | Toddlers should be allowed to eat fast food to make them happy | Para que as crianças pequenas fiquem felizes, deve-se permitir que elas comam fast food (comida pronta rápida servida em lanchonetes, barracas de cachorro quentes, pizzaria...). | Para que as crianças pequenas fiquem felizes, deve-se permitir que elas comam fast food (comida pronta rápida servida em lanchonetes, barracas de cachorro quentes, pizzaria...).. |  |
| ID31 | As crianças pequenas devem ter permissão para beber bebidas açucaradas/refrigerantes, para que fiquem felizes | Crianças pequenas devem ter permissão de beber bebidas açucaradas para que fiquem felizes | Crianças pequenas deveriam poder beber bebidas açucaradas/refrigerantes para que fiquem felizes | Small children should be able to drink sugary drinks/soda to keep them happy | Toddlers should be allowed to drink sugary/soft drinks to make them happy | Para que as crianças pequenas fiquem felizes, deve-se permitir que elas tomem bebidas açucaradas (como suco de caixinha, suco em pó, água de coco de caixinha, xaropes de guaraná/groselha, suco de fruta com adição de açúcar)/refrigerantes. | Para que as crianças pequenas fiquem felizes, deve-se permitir que elas tomem bebidas açucaradas (como suco de caixinha, suco em pó, água de coco de caixinha, xaropes de guaraná/groselha, suco de fruta com adição de açúcar)/refrigerantes |  |
| ID32 | As crianças pequenas devem ter permissão para comer sobremesas/doces, para que fiquem felizes | Crianças pequenas deveriam poder comer sobremesas/doces para que fiquem felizes | Crianças pequenas deveriam poder comer sobremesas/doces para que fiquem felizes | Small children should be able to eat dessert/sweets to keep them happy | Toddlers should be allowed to eat dessert/candy to make them happy | Para que as crianças pequenas fiquem felizes, deve-se permitir que elas comam sobremesas/doces. | Para que as crianças pequenas fiquem felizes, deve-se permitir que elas comam sobremesas/doces. |  |

^a^ The cross-cultural adaptation was based on the itens from the Infant Feeding Style Questionnaire (IFSQ) (Thompson et al., 2009).
