# Supplementary material for: Cross-cultural adaptation and validation of the Infant Feeding Style Questionnaire in Brazil
Source: PLoS One. 2021 Sep 30;16(9):e0257991. doi: 10.1371/journal.pone.0257991 (PMC8483293; doi:10.1371/journal.pone.0257991)
Supplement: S1 Questionnaire — (DOCX) [file pone.0257991.s003.docx]

| Item ^a^ | IFSQ-Br |
| --- | --- |
| Feeding style: Laissez-Faire | **Estilo de alimentação: Laissez-Faire** |
| Attention | **Atenção** |
| LF1 | Quando o/a (nome da criança) toma/tomava mamadeira, eu seguro/segurava (a mamadeira) para cima |
| LF2 | O/A (nome da criança) assiste TV enquanto come |
| LF3 | Eu assisto TV enquanto dou comida para o/a (nome da criança) |
| LF4 | Eu não vejo problema em segurar a mamadeira para os bebês (enquanto eles estão mamando) |
| LF5 | Não tem problema deixar crianças pequenas ficarem andando enquanto comem, desde que elas comam |
| Diet quality | **Qualidade da dieta** |
| LF6 | Eu monitoro o que o/a (nome da criança) come |
| LF7 | Eu monitoro a quantidade de comida que o/a (nome da criança) come |
| LF8 | Eu fico atenta para que o/a (nome da criança) não coma alimentos açucarados, como balas, sorvetes, bolos ou biscoitos |
| LF9 | Eu fico atenta para que o/a (nome da criança) não coma "besteiras" ou "porcarias", como batatas fritas de pacote ou outros salgadinhos de pacote |
| LF10 | Crianças pequenas deveriam poder comer o que quiserem nos lanches |
| LF11 | Crianças pequenas deveriam poder comer o que quiserem quando estiverem comendo na rua ou fora de casa |
| Feeding style: Pressuring | **Estilo de alimentação: Pressionador** |
| Finish | **Término** |
| PR1 | Eu tento fazer com que o/a (nome da criança) termine de comer toda a sua comida |
| PR2 | Mesmo se o/a (nome da criança) parecer cheio (a), incentivo a comer tudo mesmo assim |
| PR3 | Eu tento fazer com que o/a (nome da criança) termine a mamada do peito ou o leite da mamadeira (fórmula) |
| PR4 | Eu tento fazer com que o/a (nome da criança) coma mesmo se ele/ela estiver sem fome |
| PR5 | Eu insisto/tento novamente  servir um alimento novo que o/a (nome da criança) não quis naquela mesma refeição |
| PR6 | Eu elogio a cada colherada para incentivar o/a (nome da criança) a comer tudo |
| PR7 | É importante que crianças pequenas comam tudo que está no prato |
| PR8 | É importante que bebês terminem de tomar todo o leite da mamadeira |
| Cereal | **Cereais** |
| PR11 | Eu dou/dava ao/à (nome da criança) cereais engrossantes (como amido de milho e farinhas) na mamadeira |
| PR12 | Cereais engrossantes (como amido de milho e farinhas) na mamadeira ajudam os bebês a dormirem a noite toda |
| PR13 | Colocar cereais engrossantes (como amido de milho e farinhas) na mamadeira é bom pois ajuda os bebês a se sentirem cheios |
| PR14 | Bebês com menos de 6 meses precisam de outros alimentos além do leite materno ou leite da mamadeira (fórmula) para se sentirem cheios |
| PR15 | Bebês com menos de 6 meses precisam de outros alimentos além do leite materno ou leite da mamadeira (fórmula) para dormirem a noite toda |
| Soothing | **Acalmando** |
| PR16 | Quando o/a (nome da criança) chora, eu o/ a alimento imediatamente |
| PR17 | A melhor forma de fazer bebês pararem de chorar é alimentando-os |
| PR18 | A melhor forma de fazer crianças pequenas pararem de chorar é dando comida a elas |
| PR19 | Quando os bebês choram, geralmente significa que estão com fome |
| Feeding style: Restrictive | **Estilo de alimentação: Restritivo** |
| Amount | **Quantidade** |
| RS1 | Eu controlo com cuidado a quantidade de comida que o/a (nome da criança) come |
| RS2 | Eu tomo bastante cuidado para não alimentar o/a (nome da criança) demais/em excesso |
| RS3 | É importante que os pais tenham regras sobre a quantidade de comida que as crianças pequenas devem comer |
| RS4 | É importante que os pais decidam a quantidade de comida que os bebês devem comer |
| Diet quality | **Qualidade da alimentação** |
| RS5 | Eu deixo o/a (nome da criança) comer fast food (comida pronta rápida servida em lanchonetes, barracas de cachorro quentes, pizzaria...). |
| RS6 | Eu deixo o/a (nome da criança) comer “besteiras” ou "porcarias" (como salgadinho de pacote, balinhas, biscoitos recheados...) |
| RS7 | Crianças pequenas nunca devem comer fast food (comida pronta rápida servida em lanchonetes, barracas de cachorro quentes, pizzaria...) |
| RS8 | Bebês nunca devem comer fast food (comida pronta rápida servida em lanchonetes, barracas de cachorro quentes, pizzaria...) |
| RS9 | Crianças pequenas nunca devem comer alimentos açucarados, como biscoitos recheados |
| RS10 | Crianças pequenas nunca devem comer “besteiras” ou "porcarias", como salgadinhos de pacote |
| RS11 | Crianças pequenas devem comer apenas alimentos e comidas saudáveis |
| Feeding style: Responsive | **Estilo de alimentação: Responsivo** |
| Satiety | **Saciedade** |
| RP1 | O/A (nome da criança) me mostra quando ele/ela está cheio (a) |
| RP2 | O/A (nome da criança) me mostra quando ela/ele está com fome |
| RP3 | Eu deixo o/a (nome da criança) decidir o quanto vai comer |
| RP4 | Eu presto atenção quando o/a (nome da criança) parece me dizer que ele/ela está cheio (a) ou com fome |
| RP5 | Eu permito que o/a (nome da criança) coma quando ele/ela está com fome |
| RP6 | As crianças sabem quando elas estão cheias |
| RP7 | As crianças sabem quando estão com fome e precisam comer |
| Attention | **Atenção** |
| RP8 | Eu converso com o/a (nome da criança) para incentivá-lo (a) a tomar mamadeira (fórmula)/ou mamar no peito |
| RP9 | Eu converso com o/a (nome da criança) para incentivá-lo (a) a comer |
| RP10 | Eu mostro para o/a (nome da criança) como comer dando uma mordida ou fingindo morder |
| RP11 | Eu tento oferecer novamente ao/à (nome da criança) alimentos que ele /ela rejeitou quando ofereci na primeira vez |
| RP12 | É importante ajudar ou incentivar crianças pequenas a comerem |
| Feeding style: Indulgence | **Estilo de alimentação: Indulgente** |
| Permissive | **Permissivo** |
| ID1 | Eu permito que o/a (nome da criança) assista TV enquanto come caso ele/ela queira |
| ID2 | Eu permito que o/a (nome da criança) coma fast food (comida pronta rápida servida em lanchonetes, barracas de cachorro quentes, pizzaria...) caso ele/ela queira |
| ID3 | Eu permito que o/a (nome da criança) tome bebidas açucaradas (como suco de caixinha, suco em pó, água de coco de caixinha, xaropes de guaraná/groselha, suco de fruta com adição de açúcar)/refrigerantes caso ele/ela queira |
| ID4 | Eu permito que o/a (nome da criança) coma sobremesa/doces caso ele/ela queira |
| ID5 | Se as crianças pequenas quiserem, deve-se permitir que elas assistam TV enquanto comem. |
| ID6 | Se as crianças pequenas quiserem, deve-se permitir que elas comam fast food (comida pronta rápida servida em lanchonetes, barracas de cachorro quentes, pizzaria...) |
| ID7 | Se as crianças pequenas quiserem, deve-se permitir que elas tomem bebidas açucaradas(como suco de caixinha, suco em pó, água de coco de caixinha, xaropes de guaraná/groselha, suco de fruta com adição de açúcar)/ refrigerantes. |
| ID8 | Se as crianças pequenas quiserem, deve-se permitir que elas comam sobremesas/doces. |
| Coaxing | **Persuadindo** |
| ID9 | Eu permito que o/a (nome da criança) assista TV enquanto come para garantir que ele/ela coma o suficiente |
| ID10 | Eu permito que o/a (nome da criança) coma fast food (comida pronta rápida servida em lanchonetes, barracas de cachorro quentes, pizzaria...) para garantir que ele/ela coma o suficiente |
| ID11 | Eu permito que o/a (nome da criança) tome bebidas açucaradas (como suco de caixinha, suco em pó, água de coco de caixinha, xaropes de guaraná/groselha, suco de fruta com adição de açúcar)/refrigerantes para garantir que ele/ela beba o suficiente |
| ID12 | Eu permito que o/a (nome da criança) coma sobremesas/doces para garantir que ele/ela coma o suficiente |
| ID13 | Para garantir que as crianças pequenas comam o suficiente, deve-se permitir que elas assistam TV enquanto comem. |
| ID14 | Para garantir que as crianças pequenas comam o suficiente, deve-se permitir que elas comam fast-food (comida pronta rápida servida em lanchonetes, barracas de cachorro quentes, pizzaria...) |
| ID15 | Para garantir que as crianças pequenas bebam o suficiente, deve-se permitir que elas tomem bebidas açucaradas (como suco de caixinha, suco em pó, água de coco de caixinha, xaropes de guaraná/groselha, suco de fruta com adição de açúcar)/refrigerantes |
| ID16 | Para garantir que as crianças pequenas comam o suficiente, deve-se permitir que elas comam sobremesas/doces. |
| Soothing | **Acalmando** |
| ID17 | Eu permito que o/a (nome da criança) assista TV enquanto come para evitar que ele/ela chore |
| ID18 | Eu permito que o/a (nome da criança) coma fast food (comida pronta rápida servida em lanchonetes, barracas de cachorro quentes, pizzaria...) para evitar que ele/ela chore |
| ID19 | Eu permito que o/a (nome da criança) tome bebidas açucaradas (como suco de caixinha, suco em pó, água de coco de caixinha, xaropes de guaraná/groselha, suco de fruta com adição de açúcar)/ refrigerantes para evitar que ele/ela chore |
| ID20 | Eu permito que o/a (nome da criança) coma sobremesa/doces para evitar que ele/ela chore |
| ID21 | Para evitar que crianças pequenas chorem, deve-se permitir que elas assistam TV enquanto comem. |
| ID22 | Para evitar que crianças pequenas chorem, deve-se permitir que elas comam fast food (comida pronta rápida servida em lanchonetes, barracas de cachorro quentes, pizzaria...) |
| ID23 | Para evitar que crianças pequenas chorem, deve-se permitir que elas tomem bebidas açucaradas (como suco de caixinha, suco em pó, água de coco de caixinha, xaropes de guaraná/groselha, suco de fruta com adição de açúcar)/ refrigerantes. |
| ID24 | Para evitar que crianças pequenas chorem, deve-se permitir que elas comam sobremesas/doces. |
| Pampering | **Mimando** |
| ID25 | Eu permito que o/a (nome da criança) assista TV enquanto come para deixá-lo (a) feliz |
| ID26 | Eu permito que o/a (nome da criança) coma fast food (comida pronta rápida servida em lanchonetes, barracas de cachorro quentes, pizzaria...) para deixá-lo (a) feliz |
| ID27 | Eu permito que o/a (nome da criança) tome bebidas açucaradas (como suco de caixinha, suco em pó, água de coco de caixinha, xaropes de guaraná/groselha, suco de fruta com adição de açúcar)/ refrigerantes para deixá-lo (a) feliz |
| ID28 | Eu permito que o/a (nome da criança) coma sobremesa/doces para deixá-lo (a) feliz |
| ID29 | Para que as crianças pequenas fiquem felizes, deve-se deve-se permitir que elas assistam TV enquanto comem. |
| ID30 | Para que as crianças pequenas fiquem felizes, deve-se permitir que elas comam fast food (comida pronta rápida servida em lanchonetes, barracas de cachorro quentes, pizzaria...).. |
| ID31 | Para que as crianças pequenas fiquem felizes, deve-se permitir que elas tomem bebidas açucaradas (como suco de caixinha, suco em pó, água de coco de caixinha, xaropes de guaraná/groselha, suco de fruta com adição de açúcar)/refrigerantes |
| ID32 | Para que as crianças pequenas fiquem felizes, deve-se permitir que elas comam sobremesas/doces. |
